# Improving evidence-based grouping of transitional care strategies in hospital implementation using statistical tools and expert review

**DOI:** 10.1186/s12913-020-06020-9

**Published:** 2021-01-07

**Authors:** Jing Li, Gaixin Du, Jessica Miller Clouser, Arnold Stromberg, Glen Mays, Joann Sorra, Jane Brock, Terry Davis, Suzanne Mitchell, Huong Q. Nguyen, Mark V. Williams

**Affiliations:** 1 Center for Health Services Research, University of Kentucky, Lexington, USA; 2 Department of Statistics, College of Arts and Sciences, University of Kentucky, Lexington, USA; 3 Colorado School of Public Health, University of Colorado Anschutz, Aurora, USA; 4 Westat, Rockville, USA; 5 Telligen Quality Improvement Organization, West Des Moines, USA; 6 Louisiana State University, Baton Rouge, USA; 7 Boston Medical Center, Boston University, Boston, USA; 8 Kaiser Permanente, Oakland, USA

**Keywords:** Transitional care, Hospital readmissions, Patient-centeredness

## Abstract

**Background:**

As health systems transition to value-based care, improving transitional care (TC) remains a priority. Hospitals implementing evidence-based TC models often adapt them to local contexts. However, limited research has evaluated which groups of TC strategies, or transitional care activities, commonly implemented by hospitals correspond with improved patient outcomes. In order to identify TC strategy groups for evaluation, we applied a data-driven approach informed by literature review and expert opinion.

**Methods:**

Based on a review of evidence-based TC models and the literature, focus groups with patients and family caregivers identifying what matters most to them during care transitions, and expert review, the Project ACHIEVE team identified 22 TC strategies to evaluate. Patient exposure to TC strategies was measured through a hospital survey (*N* = 42) and prospective survey of patients discharged from those hospitals (*N* = 8080). To define groups of TC strategies for evaluation, we performed a multistep process including: using ACHIEVE’S prior retrospective analysis; performing exploratory factor analysis, latent class analysis, and finite mixture model analysis on hospital and patient survey data; and confirming results through expert review. Machine learning (e.g., random forest) was performed using patient claims data to explore the predictive influence of individual strategies, strategy groups, and key covariates on 30-day hospital readmissions.

**Results:**

The methodological approach identified five groups of TC strategies that were commonly delivered as a bundle by hospitals: 1) *Patient Communication and Care Management*, 2) *Hospital-Based Trust, Plain Language, and Coordination*, 3) *Home-Based Trust, Plain language, and Coordination*, 4) *Patient/Family Caregiver Assessment and Information Exchange Among Providers*, and 5) *Assessment and Teach Back*. Each TC strategy group comprises three to six, non-mutually exclusive TC strategies (i.e., some strategies are in multiple TC strategy groups). Results from random forest analyses revealed that TC strategies patients reported receiving were more important in predicting readmissions than TC strategies that hospitals reported delivering, and that other key co-variates, such as patient comorbidities, were the most important variables.

**Conclusion:**

Sophisticated statistical tools can help identify underlying patterns of hospitals’ TC efforts. Using such tools, this study identified five groups of TC strategies that have potential to improve patient outcomes.

**Supplementary Information:**

The online version contains supplementary material available at 10.1186/s12913-020-06020-9.

## Background

More than 25 million people experience a hospital inpatient stay at U.S. hospitals during the course of a year [1]. Subsequent transitions from hospital to home and community settings are too often uncoordinated, disruptive, and costly [2–4]. Unplanned hospital readmissions represent an important consequence of poorly managed care transitions, resulting in poorer patient experience, elevated risks of hospital-acquired conditions and injury, and more than $25 billion annually in healthcare costs [5]. During the past decade, the U.S. government invested billions of dollars in demonstration programs, quality improvement initiatives, and value-based payment incentives designed to improve care transitions and reduce unplanned hospital readmissions [6, 7]. Specifically, the U.S. Centers for Medicare and Medicaid Services (CMS) supported organized, evidence-based TC programs such as Project RED (Re-Engineering Discharge), [8] Project BOOST (Better Outcomes by Optimizing Safe Transitions, [9] the Care Transitions Intervention (CTI), [10] and the Transitional Care Model (TCM) [11]. Encouraging signs of progress emerged in recent years, including a growing collection of research-tested interventions to improve care transitions, [7, 10, 12, 13] and a downward trend in readmission rates among Medicare beneficiaries [14–16]. This progress has been uneven among U.S. hospitals and the extent disputed, [17] with wide variation in readmission rates and persistently elevated rates among low-income patients and other vulnerable subgroups at safety net hospitals [18, 19]. Notably, all the various evidence-based TC programs are characterized by multiple components, share some interventions and have some unique ones.

Uneven progress in improving care transitions may result at least in part from variation in the practices adopted and implemented by hospitals to manage care transitions. A 2010 survey of hospitals participating in a national quality improvement initiative for care transitions found that the average hospital implemented less than half of the 10 recommended practices, such as alerting outpatient physicians about patient discharges within 48 h (39% of hospitals), sending a discharge summary to the patient’s primary physician (23% of hospitals), and following up on test results that returned after patients are discharged (34% of hospitals) [20]. A follow-up study of these same hospitals in 2012 found increases in hospitals’ use of some practices, but no change in use of others [21]. Hospitals often provide multiple care transition interventions to patients, sometimes selecting components from several different models based on staff knowledge, preferences, and expectations about feasibility and effectiveness [22, 23]. Little systematic evidence currently exists about the combinations of care transition practices that hospitals group together and implement. Identifying and understanding which practices hospitals use and which they group to deliver as a bundle aimed at improving care transitions are necessary first steps for evaluating the comparative effectiveness of varying approaches to care transitions experienced by patients as they go through the hospital discharge process.

**Fig. 1:**
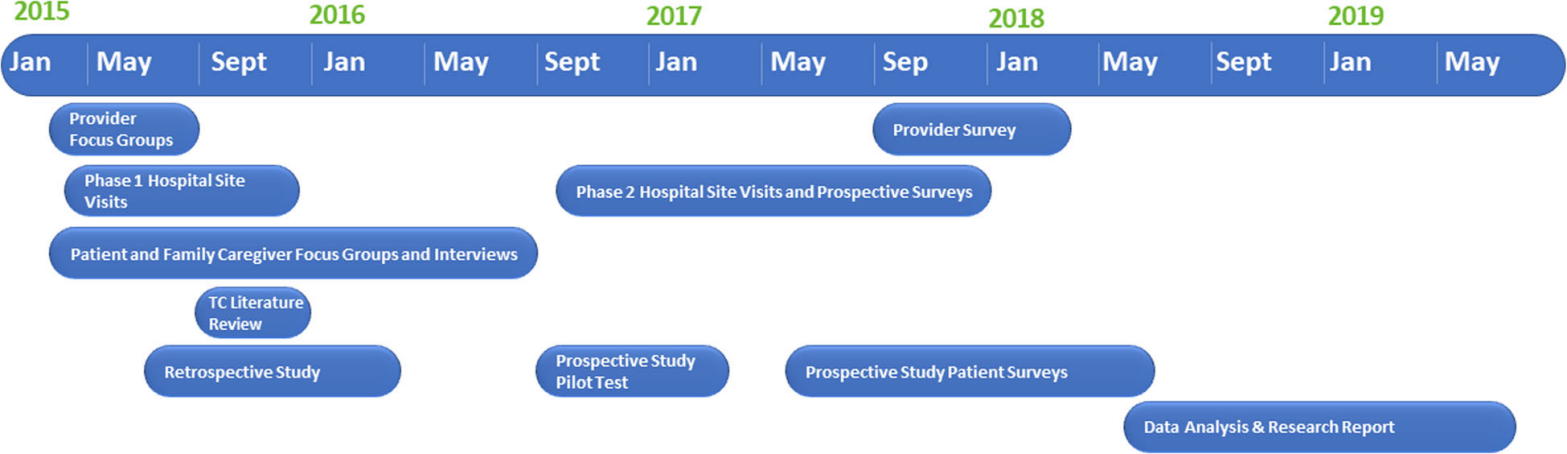
Project ACHIEVE Components and Timeline (attached file)

Project ACHIEVE (Achieving Patient-Centered Care and Optimized Health In Care Transitions by Evaluating the Value of Evidence) [24] was funded by the Patient-Centered Outcomes Research Institute (PCORI®) to convene patients and family caregivers with nationally recognized healthcare researchers to identify which TC services and outcomes matter most to patients and family caregivers [25] and to rigorously evaluate ongoing efforts seeking to improve care transitions. This 5-year multi-component, mixed methods project in the U.S. involved a research team with principal investigators from three academic medical centers, an integrated health system and a large survey company, guided by a Stakeholder Advisory Group and a Scientific Advisory Committee. The research team included experts in health services research, survey methodology, qualitative research, care transitions, health literacy, family caregivers, implementation science, and organizational behavior. This complex initiative involved multiple simultaneous components carried out at different times as shown in Fig. 1. One of the specific aims of ACHIEVE was to determine which evidence-based transitional care (TC) strategies or groups of them most effectively yield patient and caregiver desired outcomes. An essential step in this process was identifying which groups of TC strategies were commonly delivered as a bundle by hospitals. We report here the methodology used to identify and define combinations of transitional care strategies, or groups of activities, implemented among a large and diverse cohort of U.S. short-term acute-care hospitals that aimed to improve an array of patient outcomes. The primary goal of this manuscript is to describe this methodology by which the Project ACHIEVE research team utilized information from multiple components of Project ACHIEVE to determine the groupings of TC strategies with relevant relationship to 30-day readmissions and patient reported outcomes.

## Methods/design

### Study design

The methodology presented in this manuscript informed one of the specific aims of the larger Project ACHIEVE [24]. Project ACHIEVE is an observational study with the overall aims of identifying what matters most to patients and family caregivers during care transitions, and identifying which combinations of TC strategies yield desired outcomes for diverse groups of patients and family caregivers. ACHIEVE evaluated the natural experiment [26, 27] being conducted in the U.S. regarding the selective implementation of evidence-based transitional care models to accomplish this overall aim. Of note, we define TC Strategies as TC practices or efforts that aim to improve care transitions; such TC strategies are often bundled together by hospitals (e.g., Projects RED or BOOST). Our methodology called for identifying and defining a priori groups of TC strategies commonly delivered as bundles by hospitals. This process included survey of hospital employees actively engaged in care transitions; survey of Medicare beneficiaries discharged from hospitals; use of exploratory factor analysis, latent class analysis, and finite mixture model; and iterative review by research team members, many of whom are nationally recognized experts in care transitions research. The process was informed by ACHIEVE’S prior retrospective analysis of 2.4 million Medicare beneficiaries across 5 years [28]. These a priori defined groups of TC strategies are delineated and defined in Tables 3, 4. Detailed explanation as to how they were derived is provided in the TC Strategy Development section of the Methods section.

### Setting

Forty short-term acute care hospitals and two critical access hospitals in the U.S.

### Participants

Medicare beneficiaries discharged from hospitals that participated in Project ACHIEVE’s prospective study were eligible to be participants. Using a purposive sampling strategy, hospitals were recruited by the research team and its partners to ensure diversity in: 1) urbanicity; 2) safety-net status; 3) critical access status; 4) member of an integrated delivery system; and at least some participation in 5) alternative payment models, and/or 6) formal evidence-based TC programs (e.g., Project RED, Re-engineering Discharge; Project BOOST, Better Outcomes by Optimizing Safe Transitions) or community-based TC programs (e.g., Centers for Medicare and Medicaid Services’ Community-based Care Transitions Program – CMS CCTP). A hospital recruitment email was sent by ACHIEVE partners (American Hospital Association, America’s Essential Hospitals, Joint Commission Resources) to hospitals known to have a formal TC effort through their participation in one of the following programs: CCTP; Quality Improvement Organization Integrating Care for Populations and Communities (QIO ICPC); and Hospital Engagement Network (HEN), which became the Hospital Innovation Improvement Networks (HIINs) in 2016. The ACHIEVE team followed up with interested hospitals and confirmed participation.

#### Hospital survey

Representatives from all 42 hospitals agreeing to participate in Project ACHIEVE completed a cross-sectional, web-based survey (See Additional file 1: Appendix 1; it is not under license), to examine their hospital’s implementation of TC efforts from October 2016 through December 2017. Project ACHIEVE hospital coordinators at each site were nominated by hospitals based on their participation in implementing TC strategies in the hospital. These staff were sent web-based survey links through REDCap, [29] a HIPAA-compliant survey platform. The overlapping roles of responding staff included quality improvement (38%), case management/care coordination/social work (52%), patient safety (5%), clinical role—e.g., physician or nurse (14%), other non-clinical (7%), or roles such as research or project management staff (12%). Survey content was generated by the research team and informed by an updated review of available literature, [30] ACHIEVE patient and family caregiver focus groups, [25] input from the project’s Scientific Advisory Council (SAC) and Stakeholder Advisory Group (SAG), ACHIEVE’s Phase 1 hospital site visits, [23] and was revised based on results from ACHIEVE’s retrospective study which also included a hospital survey [28]. Respondents also reported on the range of organized TC programs supported by CMS—e.g., including Project RED, [8] Project BOOST, [31] Coleman’s Care Transitions Initiative, [12] and Naylor’s Transitional Care Model [32]—and indicated whether or not their organization implemented specific TC practices or strategies included in evidence-based care transition models and/or recommended by professional and scientific organizations. Research staff validated survey data through follow up phone calls to staff or through subsequent 1 to 2 day, in-person site visits with various transitional care stakeholders from the hospital and its patient community.

Hospital survey data were linked with corresponding records from the 2017 American Hospital Association (AHA) annual survey and the 2018 CMS Impact File (Table 1) to obtain information on hospital facility characteristics, including number of staffed beds, ownership, and teaching status.

**Table 1:** Characteristics of Study Hospitals Compared to National Samples

	Study Hospitals ***N*** = 42		AHA Hospitals^**a**^ ***N*** = 4700	
Characteristic	N	(%)	N	(%)
**Region**				
Midwest	8	19%	1396	30%
Northeast	9	21%	571	12%
Puerto Rico	0	0%	50	1%
South	8	19%	1762	37%
West	17	40%	921	20%
Unknown	0	0	5	0.1%
**Total Licensed Beds**				
< 100	6	14%	1750	37%
100–299	12	29%	1191	25%
≥ 300	24	57%	909	19%
Unknown	0	0%	855	18%
**Organizational Control**				
Government, nonfederal	8	19%	1018	22%
Nongovernment, non- profit	33	79%	2764	59%
Investor-owned, for-profit	1	2%	886	19%
Government, federal	0	0%	37	0.8%
	**Study Hospitals** **N = 42**		**CMS Hospitals** ***N*** **= 3331**	
	**N**	**%**	**N**	**%**
**Teaching Status**				
Major Teaching	16	38%	382	11%
Minor Teaching	21	50%	1316	40%
Non-teaching	5	12%	1633	49%
**Urban/Rural Status**^**b**^				
Large urban	25	60%	3452	55%
Other urban	8	19%	1909	31%
Rural	9	21%	890	14%

^a^ 2017 AHA hospital survey, acute/critical care only

^b^ from Fiscal Year 2018 CMS Impact File

Characteristics of the 42 participating hospitals are available in Table 1. Compared to the 2017 nationally representative American Hospital Association Survey, [33] ACHIEVE hospitals were more often in the West (40% vs. 20%), had at least 300 beds (57% vs 19%), were teaching (88% vs. 51%), and nongovernment, non-profit (79% vs. 59%); 13 of the 42 (31%) hospitals were members of one large regional integrated health system (Kaiser).

### Patient survey

We developed the post-hospital discharge patient survey to assess: patient perception of receipt of TC strategies received at the hospital or since being home, care experience, patient-reported health outcomes, caregiver effort and stress, and background and demographic questions. We used existing validated instruments when possible, such as items in the NIH PROMIS [34] repository, but most items derived from constructs elicited from study focus groups/interviews [25] and hospital site visits [23]. Survey questions and response options were evaluated through cognitive interviews involving 34 patients and 34 family caregivers. The patient survey contained assessment of 11 TC strategies, 5 of which were also assessed in the above hospital survey. While both sources of data (hospital and patient) were used in the analytic process to define TC strategy groups, in the final prospective analysis, we used only one data source for each strategy. Ultimately, the hospital survey was used for (*n* = 16 strategies), due to the targeted nature of many TC efforts (e.g., community service referrals). We assessed 6 of the final 22 TC strategies through the patient survey, as they were viewed as being most reliably measured from the patient perspective (e.g., plain language communication). Survey instruments were revised based on these findings from the earlier Project ACHIEVE study components and extensive conversations among the research team, Stakeholder Advisory Group (SAG) and Scientific Advisory Council (SAC). The updated instruments were pilot-tested during a five-month period among five participating hospitals. Based on pilot findings, and again with input from the research team, SAG and SAC, the surveys were further refined. The patient survey is provided in Additional file 2: Appendix 2; it is not under license.

Patient recruitment occurred in hospitals from June 2017 to April 2018. Adult participants were eligible if they were hospitalized on the medical or surgical units at the participating hospitals. For all non-Kaiser hospitals (*n* = 29), inclusion criteria required patients to have traditional Medicare Fee for Service (FFS); for Kaiser hospitals (*n* = 13), inclusion criteria included patients with Medicare Advantage or FFS. Exclusion criteria included: 1) in-hospital death, 2) transferred to another acute-care hospital, 3) discharged against medical advice, 4) admission for primary diagnosis of psychiatric condition, rehabilitation, or medical treatment of cancer; 5) current prisoner; or 6) currently under suicide watch. Participating hospital staff identified eligible patients during their hospitalization and obtained HIPAA authorization and contact information for those interested in being contacted to complete a mail or phone survey about their TC experiences in the hospital and at home. Recruited patients were mailed survey packets (in English or Spanish) beginning 51 days post-discharge as per CMS requirements to avoid conflicts with Hospital Consumer Assessment of Healthcare Providers and Systems (HCAHPS) surveys. Non-responders received a reminder after 7 days, a second survey after 24 days, a phone call after 34 days; and were retired after up to 5 call attempts.

**Fig. 2:**
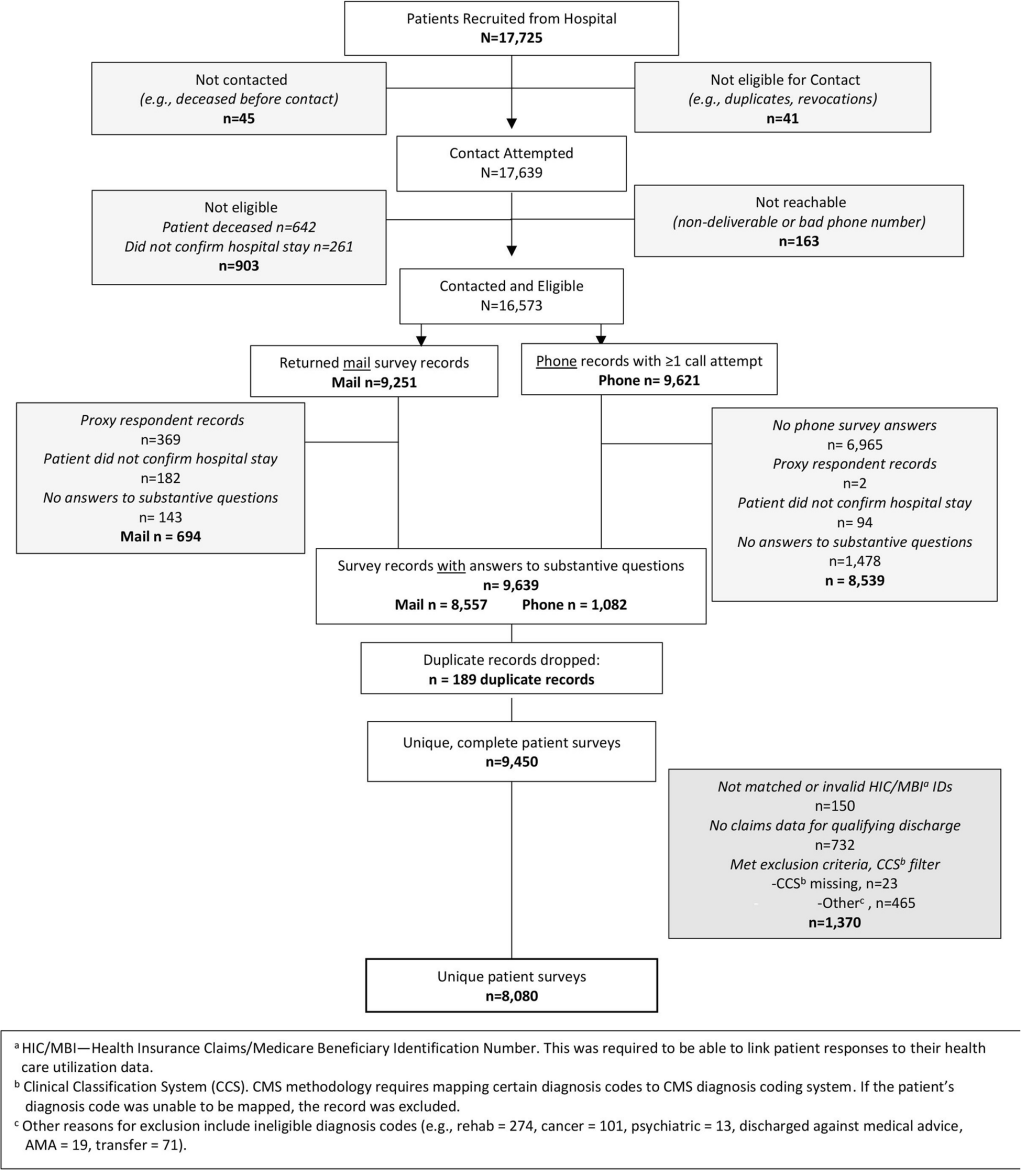
Patient Sample Flow Chart (attached file)

From July 2017 to July 2018, 57 % of recruited patients (*N* = 17,639) responded to the survey. On average, patients completed surveys 75 days post-discharge (Range 52–259). See Fig. 2 for patient survey flow chart. Ultimately, 8080 patients were included in the process of identifying TC strategy groups with a mean age of 72.3 (SD = 10.1). Consistent with the 2017 Medicare population [35], of which 54% were female and 76% were White, a majority of participants were female (53.6%), White (78.9%), and were eligible for Medicare due to their age (78.9%). See Table 2.

**Table 2:** Patient Characteristics, N = 8080

	N	%
**Age** Mean (SD)	72.3	10.1
**Gender**		
Female	4333	53.6%
Male	3747	46.4%
Total	8080	100%
**Race**		
Unknown	350	4.3%
Other	523	6.5%
Black	833	10.3%
White	6374	78.9%
Total	8080	100%
**Medicare Eligibility**		
Age	6377	78.9%
Disability	949	11.7%
ESRD	225	2.8%
SLMB	506	6.26%
**Dual Eligible**		
Yes	1260	15.59%

Notes

ESRD = End Stage Renal Disease

SLMB = Specified Low-Income Medicare Beneficiary

### TC strategy development

**Fig. 3:**
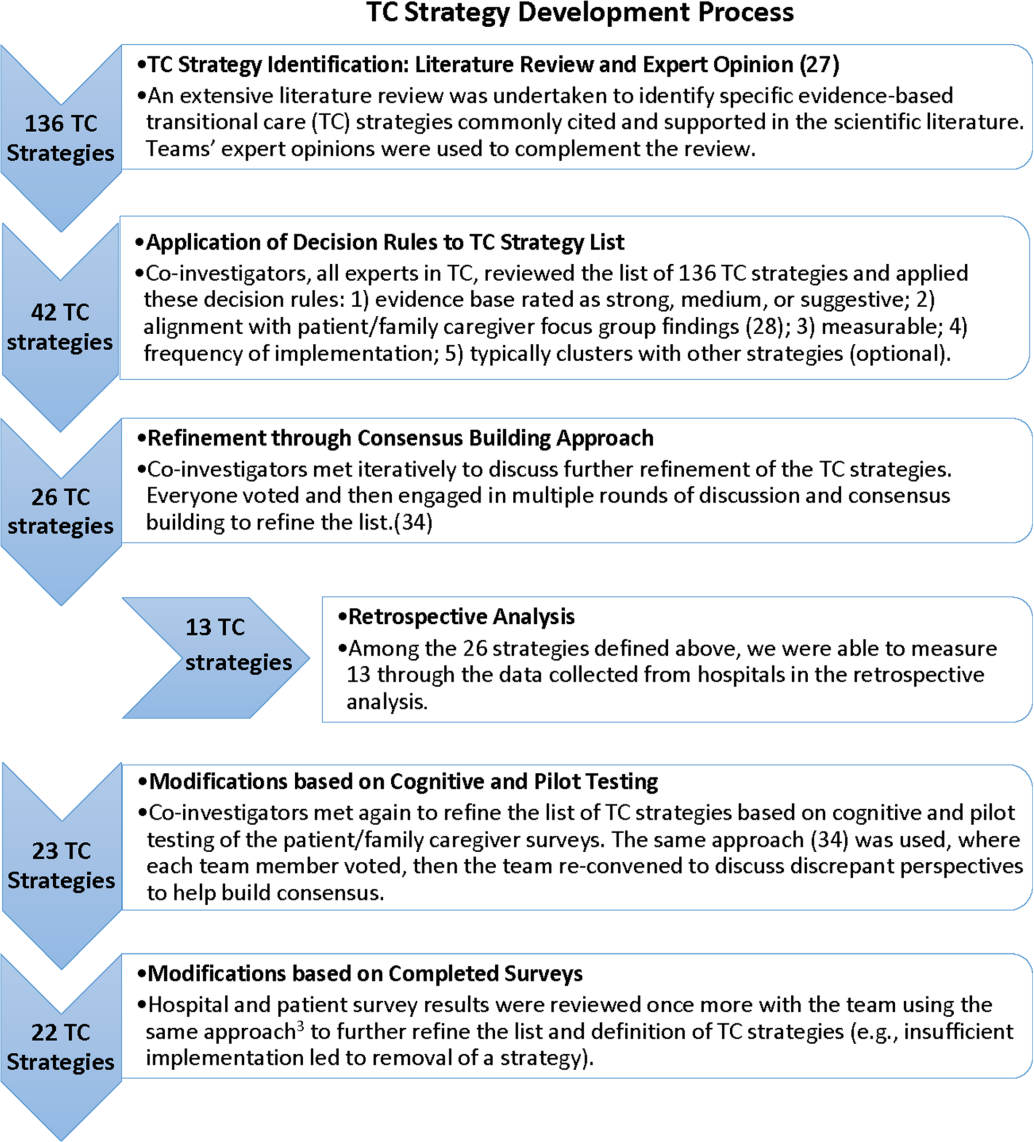
TC Strategy Development Process (attached file)

The selection of individual TC strategies to be evaluated in Project ACHIEVE, and their definitions, resulted from a multistep process including: 1) an extensive review of the TC literature, [30] 2) focus groups and interviews conducted with nearly 250 patients and family caregivers, [25] 3) a retrospective analysis of TC strategies at 370 hospitals, [28] 4) input and feedback from the study’s Scientific Advisory Council (SAC) and the Stakeholder Advisory Group (SAG), and 5) through iterative conversations among the research team’s investigators which included experts and leaders in transitional care and research methodology using a modified Delphi process aiming to identify the most important ones that could be reasonably evaluated through surveys of hospitals and patients experiencing the hospital discharge process [36].. See Fig. 3 for a flow chart describing this multi-step, iterative process. This process identified a priori 22 important TC strategies that are variably implemented by hospitals to be evaluated in ACHIEVE’s prospective survey.

Determining whether or not a patient received a TC strategy was accomplished through two approaches (see Tables 3,4 for a list of final TC strategies, their required activities—i.e., definition—and their frequencies of implementation). Recognizing the importance of patients’ perception of receiving some TC strategies, we deemed six to be more reliably measured from the patient perspective (e.g., plain language communication), and therefore used patient survey data to measure them in the final prospective analysis. Thus, for the remaining 16 of the 22 TC strategies, we used hospital-reported data with site visit validation to evaluate implementation based on their reported provision to ‘all’ or ‘most’ patients. For example, many TC strategies implemented by hospitals are based on risk stratification (e.g., referral to community services), and therefore most patients might not report having experienced the strategy though it was applied appropriately.

**Table 3:** **Transitional Care (TC) Strategy Prevalence and Definitions**^**a**^

TC Strategy and Definition	Hospitals Adopted		Patients Exposed ^**c**^	
	n	%	n	%
1. **Identification of Caregiver** ^**b**^	**42**	**100.0%**	**7939**	**100.0%**
• Organization identifies patients’ family caregiver.				
**2. Interdisciplinary Approach** ^**b**^	**41**	**97.6%**	**7927**	**99.9%**
• Organization has a designated team that facilitates the implementation of TC efforts. • Organization uses Designated Interdisciplinary Rounds/Huddles/Meetings *and* Electronic Health Record to communicate about patients’ discharge or TC needs.				
**3. Standard Protocol** ^**b**^	**41**	**97.6%**	**7814**	**98.4%**
• Organization uses a standardized template for discharge summaries.				
**4. Transition Team**	**38**	**90.5%**	**7242**	**91.2%**
• Organization routinely uses a specific transition team (i.e., care coordination) to coordinate TC plans across hospital and post-home sites of care to a great extent or somewhat				
**5. Transition Summary for Patients and Family Caregivers**	**36**	**85.7%**	**7380**	**93.0%**
• Organization consistently provides patient-centered transition record (e.g., list of diagnoses, allergies, medications, physicians, contact information) to patients/caregivers to a great extent.				
**6. Language Assessment**	**35**	**83.3%**	**6804**	**85.7%**
• Organization consistently identifies, communicates and offers interpreter service to patients who need it to a great extent. • Organization consistently provides educational materials in the language that patients prefer, if patients are non-English speaking to a great extent or somewhat.				
**7. Medication Reconciliation**	**35**	**83.3%**	**7316**	**92.2%**
• Contacts are usually or always made with outside pharmacies and/ or primary care providers for clarifying a patient’s current medication list when needed (i.e. medication reconciliation). • A designated person is responsible for conducting medication reconciliation at discharge.				
**8. Home Visits**	**34**	**81.0%**	**6259**	**78.8%**
• Hospital or a community-based organization conducts home visits after discharge, for all, most, or some patients receiving TC services by a care coordinator or equivalent.				
**9. Patient Goal/Preference Assessment**	**33**	**78.6%**	**6240**	**78.6%**
• Organization identifies patient’s health goals and preferences.				
**10. Identify High-Risk Patients and Intervene**	**33**	**78.6%**	**6296**	**79.3%**
• Organization uses a protocol or tool to identify who is at high risk of readmission or have high-risk scenarios that could potentially results in poor outcomes. • Organization consistently uses a protocol/risk assessment tool to identify patients in need of TC services somewhat or to a great extent. • Organization uses at least 6 of the 11 criteria below to identify patients in need of TC services o Certain Diagnoses of Comorbidities o Cognitive impairment o Emotional / Psychological status (Depression, Anxiety, etc.) o History of Mental Health/Behavioral Health Issues o Lack of social support (consistent caregiver, transportation, etc.) o Language barriers o Limitations with physical functioning (e.g., frailty, deconditioning, unable to perform on ADLs) o Limited/Poor health literacy o Problems with medications (Polypharmacy and/or high-risk medication such as anticoagulants) o Socioeconomic status (e.g., financial issues, homelessness, etc.) o Substance Use (History, current use or inappropriate use of alcohol, prescriptions medications, or illicit drugs) o Use of hospital/emergency department within last 30 days o Use of hospital/emergency department within last 90 days/3 months • Organization implements risk-specific interventions tailored to a patient’s individual risk of poor outcomes or other post-discharge adverse event (e.g., referral to community services or outpatient case managers for patients with psychosocial issues) to a great extent or somewhat.				
**11. Follow-up Appointment**	**32**	**76.2%**	**6100**	**76.8%**
• On the day of discharge, patients receiving TC services always or usually leave the hospital with an outpatient follow-up appointment already arranged.				
**12. Referral to Community Services**	**29**	**69.1%**	**5512**	**69.4%**
• Organization routinely make referrals and/or arrangements for community-based services to a great extent? (e.g., transportation assistance, Meals on Wheels, etc.)				
**13. Post-Discharge Care Consultation**	**27**	**64.3%**	**4868**	**61.3%**
**•** Organization regularly calls all or most patients receiving TC services after discharge to follow up on post-discharge needs or to provide additional education. • For patients discharged to skilled nursing facilities or with home health services, organization usually or always provides direct contact information for an inpatient physician to contact in case of questions.				
**14. Timely Exchange of Critical Patient Information among Providers**	**27**	**64.3%**	**5604**	**70.6%**
• There is a reliable process in place to ensure outpatient care providers (i.e., primary care physicians) are alerted to the patient’s hospital admission within 24 h of admission. • A patient’s discharge summary typically completed and available for viewing in the EMR or printed on paper either at discharge, within 48 h, or within 72 h. • For all or most patients, a paper of electronic discharge summary is sent directly to the patient’s primary care providers or post-acute providers such as nursing homes/SNFs, home health agencies, etc.? • Outpatient care and community service providers have access to all or most inpatient electronic records. • At the time of hospital discharge, goals and preferences (e.g., Goals of Care or DNR status) for all or most patients are communicated to primary care providers or post-acute providers (e.g., SNFs, home health)				
**15. Patient/Family Caregiver Transitional Care Needs Assessment**	**25**	**59.5%**	**5135**	**64.7%**
• Organization assesses patient’s TC needs using explicit criteria • Organization assesses family caregiver’s TC needs using explicit criteria • As part of the discharge process, staff or a designated person routinely asks patients whether they can afford their medications for some or all patients depending on the medications				
**16. Teach Back for Information and Skills**	**15**	**35.7%**	**2041**	**25.7%**
• Organization assesses patient’s learning capability and style • Organization formally uses the Teach Back Method [54] • Organization provides opportunities for patients and families/caregivers to learn new information or skills needed for self-care at home • Organization provides opportunities that allow patients and family/caregivers to practice new skills needed for self-care to a great extent or somewhat

**Table 4:** Transitional Care (TC) Strategy Prevalence and Definitions^d^

			Patients Exposed	
TC Strategy and Definition			n	%
**1. Plain Language Communication at Hospital**			**6214**	**78.3%**
• In the hospital, healthcare professionals explained things in a way that you could understand.				
**2. Promote Trust at Hospital**			**5777**	**72.8%**
• In the hospital, healthcare professionals definitely seemed to care about you as a person. • In the hospital, you definitely trusted healthcare professionals’ judgements about your medical care.				
**3. Helpful Health Care Contact**			**5505**	**69.3%**
• Since you’ve been home, you have had contact information for a healthcare professional you could reach out to if you had any problems or questions. • If you tried to contact them, you got help with your problems or questions or you did not try to contact them.				
**4. Plain Language Communication at Home**			**5209**	**65.6%**
• Since you’ve been home, healthcare professionals definitely explained things to you in a way you could understand.				
**5. Promote Trust at Home**			**4865**	**61.3%**
• Since you’ve been home, healthcare professionals definitely seemed to care about you as a person. • Since you’ve been home, you definitely trusted healthcare professionals’ judgements about your medical care.				
**6. Symptom Management**			**3321**	**41.8%**
• Before you left the hospital, you definitely got information about what symptoms to look out for at home. • Since you’ve been home, healthcare professionals definitely helped you manage any changes or unexpected problems with your care or you’ve had no changes or unexpected problems with your care,				
^a^ TC strategies measured through the hospital survey ^b^ Strategy was almost universally applied and therefore was not included in the final analytic groups ^c^ Patient exposure to hospital-reported strategies is calculated based on the number of patients discharged from hospitals reporting the TC strategy. ^d^ TC strategies measured through the patient survey				

### Methodology for identifying groups of TC strategies for evaluation

A multi-step process was undertaken to determine the groups of TC strategies for analysis in the prospective study, initially building upon ACHIEVE’s retrospective study [28]. A key aspect of the retrospective analysis was developing analytic approaches for the Medicare Beneficiary data files that would later be used for the prospective study reported in this manuscript. The retrospective study evaluated hospitals’ reported implementation of 13 TC strategies to determine associated changes in readmission rates and post-discharge ED utilization from 2010 to 2014. Using factor and latent class analyses, this retrospective longitudinal cohort study used survey data from 370 U.S. hospitals to classify hospitals’ TC strategy implementation into the five groups listed in Additional file 3: Appendix 3. From this retrospective study we determined analytic methodologies for identifying groups of TC strategies delivered by hospitals, and identified a group associated with the most reduction in 30-day readmissions after adjusting for the policy effect of the hospital readmission reduction penalty. We applied this methodology to an expanded number of TC strategies in the prospective study based on information gleaned from ACHIEVE focus groups conducted with patients and family caregivers [25].

### Statistical analysis

Initially, descriptive statistics were performed to measure the frequency of each TC strategy (i.e., 16 hospital-sourced and 6 patient-sourced strategies). Among the 16 strategies measured through the hospital survey, TC implementation was dichotomously coded as ‘Yes’ when all required activities were provided for ‘all’ or ‘most’ patients, and ‘No’ when they were not. Patient exposure to the hospital-sourced strategies was calculated by aggregating the number of patients discharged from all hospitals reporting ‘yes’ to the strategy. Among the 6 TC strategies measured through the patient survey, patient exposure to strategies was dichotomously coded as ‘Yes’ when all required activities were reported as ‘Yes definitely’ received by the patient^1^ and ‘No’ when they were not. See Tables 3, 4.

Three separate analyses informed the categorization of TC strategies into groups. First, we employed exploratory factor analysis (EFA) which identified the correlation structure among the TC strategies; i.e., patterns in how hospitals naturally implement strategies in bundles or groups, as well as patterns in the groups of TC strategies to which patients are exposed. Specifically, we calculated principal component (PC) estimates in factor analysis separately for the initial 11 dichotomous TC strategy variables from the patient survey (e.g., ‘Yes’ when received and ‘No’ when not; note, only 6 of these strategies were included in final groupings) and for the 16 dichotomous TC strategy variables from the hospital survey (e.g., ‘Yes’ when implemented and ‘No’ when not), each using a polychoric correlation matrix [37] and varimax rotation. The five factors whose eigenvalues exceeded one—indicating unity among variables— identified combinations of TC strategies for further analysis. The resulting factors include overlap, where individual TC strategies may load onto more than one factor, and thus, strategies may be members of more than one TC strategy group.

Next, we conducted latent class analysis (LCA) to identify unmeasured, or latent, class membership among subjects with observed variables. LCA is a nonparametric statistical method used to identify distinct but partially unobservable subgroups (classes) within a population, based on patterns of response across multiple measures [38]. Unlike factor analysis, latent class analysis identifies classes that are mutually exclusive with no overlap in TC strategies, and thus, achieves higher contrast among classes or groups than factor analytic methods. We performed LCA in order to complement our factor analysis results, in which TC strategies could overlap across different groups, thus diminishing the contrast among them. Latent class analyses were applied to TC strategy variables separately according to the source (i.e., hospital survey vs. patient survey).

Next, we used a finite mixture model (FMM) separately for hospital and patient survey data to model the probability of individual patients belonging to each unobserved group that includes different TC strategies and to classify individuals into the groups. Finite mixture modeling was conducted to determine how healthcare utilization was predicted by the selected TC strategies. It was used to further refine the TC groups as it draws inferences about how each group performs.

In addition to the analytic steps described above, the TC strategies and grouping of them were subject to expert review in the context of their conceptual or practical relevance before decisions were made regarding inclusion or exclusion of strategies in the final groups. In addition to ensuring groups with conceptual coherence and analytic significance, the team also sought to decrease overlap among groups and minimize redundancy. See Table 5 for each methodological step and its purpose.

**Table 5:** Methodological Steps for TC Strategy Group Formation

Methodological Step	Purpose of the Procedure
STEP ONE Factor Analysis	Provides an indication of how TC strategies naturally group (i.e., are implemented) together at hospitals. Resultant groups overlap, with TC strategies able to be included in multiple groups. While overlap in groups is reflective of natural practice patterns, too much overlap among groups can reduce their contrast, rendering comparative effectiveness evaluation more difficult to interpret.
STEP TWO Latent Class Analysis	Resultant groups are mutually exclusive, with no overlap in group membership for TC strategies. In addition, latent, or unmeasured characteristics are revealed among the resultant classes or groups The resultant groups from LCA provides the strength of mutual exclusivity of TC strategy membership (i.e., strategies are only grouped into one class). However, this does not reflect natural practice patterns, in which some strategies may be ubiquitous and therefore “grouped” in multiple combinations.
STEP THREE Finite Mixture Model	This step models the probability of individual patients belonging to each unobserved group of TC strategies and classifies individuals into the groups. In addition to grouping strategies based on observed traits, it draws inferences about how each group performs relative to an outcome (e.g., hospital readmissions). Thus, the total variance of each group’s contribution to readmission outcomes is also provided.
STEP FOUR Expert Review	Our research team, with experience and expertise in TC practice, research, and hospital care delivery reviewed results of the above analyses. Based on the criteria below, they determined definitions for final TC strategy groups based on these criteria. 1. Maintain consistency with TC strategy groups from the study’s retrospective analysis (Appendix 3) 2. Follow signals suggested by the analytic procedures described above 3. Ensure the conceptual relevance of TC strategies grouped together 4. Reduce overlap in TC strategies across groups to enable comparative evaluation

After the proposed groups were defined, random forest (RF) analysis**,** a supervised machine learning algorithm, [39] was conducted as a supplementary tool to investigate group composition. Random forest analyses work by creating multiple decision trees, or models, to identify the combinations among them that are influential in predicting an outcome. For the purposes of this analysis, individual TC strategies, groups of TC strategies, and key covariates (e.g., comorbidity, integrated health system affiliation, and Hierarchical Condition Category cohort^2^) were entered into a random forest analysis to ascertain their relative importance in predicting 30-day hospital readmission. Importantly, we did not use random forest to edit the TC groups’ configurations, but rather aimed to further consider our hybrid approach to identifying groups of TC strategies by examining whether random forest analysis would suggest an alternate group or method of grouping. Finally, as a form of sensitivity analysis, we ran a boosted trees analysis to determine if results were comparable to the random forest analysis. Boosted trees is a popular machine learning technique that makes predictions by combining decisions from a series of base models (decision trees or other algorithms) for a weighted average instead of simple average over a series of bootstrapped samples [40].

## Results

Apart from two TC strategies (Symptom Management and Teach Back for Information and Skills), a majority of hospitals and patients indicated implementation of or exposure to all TC strategies (Tables 3,4). Three TC strategies—Identification of Caregiver, Interdisciplinary Approach, and Standard Protocols—were implemented almost universally. Given their ubiquity, these three TC strategies were not included in any of the groups of TC strategies, as they would not help to differentiate the groups and were presumed fundamentally to be included in each TC strategy group.

Referencing ACHIEVE’s prior retrospective analysis combined with results from the exploratory factor analysis, latent class analysis, finite mixture model analysis, and expert review, our analyses supported the bundling of TC strategies into five groups of TC strategies (See Table 6): 1) *Patient Communication and Care Management*, a combination of strategies pertaining to facilitating clear and collaborative communication between providers and patients throughout the care transition from hospital to post-discharge including identification of patients’ goals or preferences and contacting patients post-discharge to address issues; 2) *Hospital-Based Trust, Plain language, and Coordination*, a combination of strategies emphasizing trust and plain language communication between providers and patients, as well as tailored care planning and pre- and post-discharge activities including medication reconciliation and contacting patients post-discharge to address issues; 3) *Home-Based Trust, Plain Language, and Coordination*, a combination of strategies emphasizing trust and plain language communication between providers and patients at the home as well as use of a specific transition team to coordinate activities including referral to community services and follow-up appointments; 4) *Patient/Family Caregiver Assessment and Information Exchange Among Providers*, assessment of transitional care needs and patients’ goals/preferences with undertaking interventions for high-risk patients combined with cross-setting information exchange among providers; and 5) *Assessment and Teach Back,* includes language assessment, use of teach back and contacting patients post-discharge to address issues. Each of these TC strategy groups comprises three to six, non-mutually exclusive TC strategies; i.e., some strategies are included in more than one TC strategy group (See Table 6). The *No TC Group* includes patients who were not exposed to any of the five TC groups, though they were exposed to other combinations of TC strategies (Range 5 to 15 TC strategies).

**Table 6:** Five Transitional Care Strategy Groups in Prospective Analysis and Requisite Strategies

TC Strategy Groups and Patients Exposed	Required TC Strategies ^a^	Methodological Steps Informing Group Membership
*Patient Communication and Care Management* (*n* = 2158, 27.2%)	• Patient Goal/Preference Assessment • Plain Language Communication at Hospital • Transition Summary for Patients and Caregivers • Helpful Health Care Contact **OR** Symptom Management • Plain Language Communication at Home • Post-Discharge Care Consultation	• Original retrospective group (*Care Plan*) • Factor analysis • Latent class analysis • Expert opinion
*Hospital-Based Trust, Plain Language, and Coordination* (*n* = 2090, 26.3%)	• Identify High-Risk Patients and Intervene • Plain Language Communication at Hospital • Promote Trust in the Hospital (care and concern expressed to patients, rapport building in the hospital) • Medication Reconciliation • Transition Summary for Patients and Caregivers • Post-Discharge Care Consultation	• Original retrospective group (*Medication Reconciliation; Care Plan*) • Factor analysis • Latent class analysis • Finite mixture model • Expert opinion
*Home-Based Trust, Plain Language, and Coordination* (*n* = 1979, 24.9%)	• Transition Team • Follow-up Appointment • Referral to Community Services • Home Visits • Promote Trust at Home (care and concern expressed to patients, rapport building post-discharge) • Plain Language Communication at Home	• Original retrospective group (*Identify High Risk*) • Latent class analysis • Finite mixture model • Expert opinion
*Patient/Family Caregiver Assessment and Information Exchange among Providers* (*n* = 3093, 39%)	• Identify High-Risk Patients and Intervene • Patient/Family Caregiver Transitional Care Needs Assessment • Patient Goal/Preference Assessment • Timely Exchange of Critical Patient Information among Providers	• Original retrospective group (*Cross-Setting Information Exchange*) • Factor analysis • Latent class analysis • Expert opinion
*Assessment and Teach Back* (*n* = 508, 6.4%)	• Language Assessment • Teach Back for Information and Skills • Post-Discharge Care Consultation	• Factor analysis • Latent class analysis • Finite mixture model • Expert opinion
No TC Group^b^ (*n* = 2042, 25.7%)	• Not in any other group	• Original retrospective group (*No TC Group*)

Note: N refers to number of patients exposed to each group; Patients may be exposed to more than one group

TC strategies throughout the tables are ordered alphabetically by care setting (e.g., first hospital-based, then bridging, then home-based

^a^ Due to their near universal application, Identification of Caregiver, Interdisciplinary Approach, and Standard Protocols are presumed to be a part of each TC group.^b^ Patients were exposed to other TC strategies, but not in the groups defined above

Factor analyses conducted with patient-derived TC strategies (*N* = 11) and those conducted with hospital-derived TC strategies (*N* = 16) each resulted in five factors (Table 7). Slightly different results were obtained from the latent class analysis which found seven classes of strategies from patient survey data and three classes of TC strategies from hospital survey data (Table 8). The findings from the finite.

**Table 7:** Factor Analysis Results

	Factor Loadings				
TC Strategies from Patient Survey	Factor 1	Factor 2	Factor 3	Factor 4	Factor 5
Plain Language Communication at the Hospital	0.47**	0.19	− 0.09	0.22*	0.00
Promote Trust at Hospital	0.69**	−0.06	0.03	0.09	0.06
Teach Back for Information and Skills ^a^	0.32**	0.36**	−0.17	0.24*	−0.05
Follow-Up Appointment ^a^	0.09	0.00	0.08	0.47**	0.04
Helpful Health Care Contact	0.02	0.06	0.15	0.18	0.46**
Post-Discharge Care Consultation ^a^	0.24*	0.32**	−0.15	0.31**	0.06
Referral to Community Services ^a^	0.01	0.01	−0.11	−0.04	0.27*
Symptom Management	−0.02	0.65**	0.06	0.05	0.10
Home Visit Received ^a^	−0.04	0.02	0.46**	0.08	−0.10
Plain Language Communication at Home	0.07	0.48**	0.45**	−0.14	0.19
Promote Trust at Home	0.35**	0.27*	0.49**	−0.23*	0.20*
**TC Strategies from Hospital Survey**	**Factor 1**	**Factor 2**	**Factor 3**	**Factor 4**	**Factor 5**
Identify High-Risk Patients and Intervene	0.82**	0.08	−0.02	0.11	0.01
Interdisciplinary Approach	0.33**	−0.14	0.57**	−0.01	−0.01
Language Assessment	0.12	0.75**	−0.15	−0.04	− 0.12
Medication Reconciliation	0.12	0.03	−0.19	−0.52**	− 0.07
Patient/Family Caregiver TC Needs Assessment	0.47**	0.11	0.09	−0.06	−0.17
Patient Goal/Preference Assessment	0.31**	−0.11	0.09	0.46**	0.21*
Plain Language at Hospital	−0.07	0.05	−0.22*	0.58**	−0.19
Standardized Protocols	−0.13	0.11	0.03	−0.20*	0.65**
Teach Back for Information and Skills	0.00	−0.64**	0.03	0.46**	−0.08
Transition Team	−0.05	0.00	0.76**	0.06	0.12
Transition Summary for Patients/Family Caregivers	0.10	0.04	−0.05	0.09	0.58**
Follow-up Appointment	0.05	0.55**	0.01	0.12	0.26*
Post-Discharge Care Consultation	−0.06	−0.32**	−0.16	0.08	0.34**
Referral to Community Services	−0.16	0.29*	0.11	0.26*	−0.05
Timely Exchange of Patient Information Among Providers	0.59**	−0.06	−0.02	− 0.33**	0.18
Home Visits	−0.05	0.02	0.61**	−0.05	−0.18

* > 0.20 ** > 0.30

Note: Although > 0.40 is standard threshold to indicate strong factor loadings, we lowered ours to 0.20 to ensure that each strategy was included in a TC group

^a^ These 5 TC strategies were measured from both the patient survey and the hospital survey. The final model used the hospital TC implementation survey as the source for these 5 strategies

^b^ Plain language communication was measured from both the hospital and patient survey; the patient survey data were ultimately used in the final model

**Table 8:** Latent Class Analysis Results

	Item Response Probability						
TC Strategies from Patient Survey	Group 1	Group 2	Group 3	Group 4	Group 5	Group 6	Group 7
**Proportion of sample within each group**	**0.42**	**0.12**	**0.11**	**0.11**	**0.10**	**0.08**	**0.07**
Plain Language Communication at Hospital ^b^	0.99^***^	0.88^*^	0.15	0.05	0.93^*^	0.05	0.90^*^
Promote Trust at Hospital	0.97^**^	0.94^*^	0.08	0.05	0.61	0.07	0.75^*^
Teach Back for Information and Skills ^a^	0.91^*^	0.41	0.31	0.90^*^	0.65	0.03	0.00
Follow-Up Appointment ^a^	0.93^*^	0.80^*^	0.80^*^	0.90^*^	0.81^*^	0.64	0.65
Helpful Health Care Contact	0.94^*^	0.76^*^	0.45	0.73	0.75^*^	0.27	0.66
Post-Discharge Care Consultation ^a^	0.97^**^	0.56	0.54	0.93^*^	0.76^*^	0.08	0.24
Referral to Community Services ^a^	0.81^*^	0.74	0.74	0.79^*^	0.71	0.61	0.73
Symptom Management	0.82^*^	0.27	0.07	0.04	0.60	0.00	0.10
Home Visits Received ^a^	0.55	0.62	0.28	0.18	0.55	0.50	0.77^*^
Plain Language Communication at Home	0.99^***^	0.86^*^	0.66	1.00^***^	0.86^*^	0.08	0.33
Promote Trust at Home	0.98^**^	0.97^**^	0.55	0.96^**^	0.35	0.12	0.26
**TC Strategies from Hospital Survey**	**Group** **1**	**Group 2**	**Group 3**				
**Proportion of sample within each group**	**0.54**	**0.29**	**0.17**				
Identify High-Risk Patients and Intervene	0.99^***^	0.43	0.72				
Interdisciplinary Approach	0.99^***^	0.91^*^	0.98^**^				
Language Assessment	0.99^***^	0.99^**^	0.06				
Medication Reconciliation	0.95^**^	0.66	0.72				
Patient/Family Caregiver TC Needs Assessment	0.73	0.43	0.45				
Patient Goal/Preference Assessment	0.77^*^	0.74	0.85^*^				
Plain Language Communication at Hospital ^b^	0.27	0.73	0.42				
Standardized Protocols	0.99^***^	0.91^*^	0.98^**^				
Teach Back for Information and Skills ^a^	0.13	0.41	0.98^**^				
Transition Team	0.86^*^	0.91^*^	0.98^**^				
Transition Summary for Patient/Family Caregivers	0.91^*^	0.75	0.85^*^				
Follow-up Appointment ^a^	0.84^*^	0.81^*^	0.43				
Post-Discharge Care Consultation ^a^	0.56	0.58	0.98^**^				
Referral to Community Services ^a^	0.61	0.91^*^	0.56				
Timely Exchange of Patient Information among Providers	0.95^**^	0.02	0.71				
Home Visits ^a^	0.74	0.89^*^	0.84^*^				

* > .75 ** > .95 *** > .99

Note: Item response probability indicates the proportion of participants in each latent class answered “yes” to each TC strategy. While there is no distinct threshold, we highlighted >.75 for the purposes of differential grouping

^a^ These 5 TC strategies were measured from both the patient survey and the hospital survey. The final model used the hospital TC implementation survey as the source for these 5 strategies

^b^ Plain language communication was measured from both the hospital and patient survey; the patient survey data were ultimately used in the final model

mixture model results using patient survey data resulted in three groups, with overall variance explained by the groups ranging from 0.012 to 0.140 (Table 9). The finite mixture model conducted with only hospital survey data showed one group (with explained variance of 0.007), including eight TC strategies. The following section details how these findings guided assignment of TC strategies to specific groups (i.e., bundles) for evaluation as interventions received by patients.

**Table 9:** Finite Mixture Model Results

TC Strategies from Patient Survey	Group 1	Group 2	Group 3
**Variance Explained by Group**	0.06	0.14	0.01
Plain Language Communication at Hospital ^b^	−0.027	−0.029	−0.006
Promote Trust at Hospital	−0.088^***^	− 0.005	− 0.006
Teach Back for Information and Skills ^a^	− 0.019	0.014	− 0.015
Follow-Up Appointment ^a^	− 0.135^***^	− 0.064^**^	− 0.051^***^
Helpful Healthcare Contact	0.028	− 0.011	0.010
Post-Discharge Care Consultation ^a^	0.008	−0.004	−0.012
Referral to Community Services ^a^	−0.036	−0.089^***^	0.004
Symptom Management	−0.069^***^	0.021	0.003
Home Visits ^a^	−0.140^***^	0.067^***^	−0.015
Plain Language Communication at Home ^b^	−0.013	−0.017	0.001
Promote Trust at Home	0.029	0.012	0.003
**TC Strategies from Hospital Survey**	**Group 1**		
**Variance Explained by Group**	.0007		
Identify High-Risk Patients and Intervene	0.017		
Language Assessment	−0.081^***^		
Medication Reconciliation	−0.053^***^		
Patient/Family Caregiver TC Needs Assessment	0.059^***^		
Patient Goal/Preference Assessment	0.023		
Plain language Communication at Hospital ^b^	0.050^***^		
Teach Back for Information and Skills	−0.003		
Transition Summary for Patients and Family Caregivers	−0.045^*^		
Follow-up Appointment ^a^	−0.037^*^		
Post-Discharge Care Consultation ^a^	0.040^***^		
Referral to Community Services ^a^	0.044^***^		
Timely Exchange of Critical Patient Information among Providers	−0.006		
Home Visits ^a^	−0.009		

Note: Low total variance explained by each group demonstrate that TC strategies alone do not contribute much to variance in readmission outcomes. In addition, in the FMM conducted with hospital survey data, plain language communication and Transition Team together in the model showed strong multi-collinearity and would not converge. Therefore, we removed Transition Team

^a^ These 5 TC strategies were measured from both the patient survey and the hospital survey. The final model used the hospital TC implementation survey as the source for these 5 strategies

^b^ Plain language communication was measured from both the hospital and patient survey; the patient survey data were ultimately used in the final model

^*^ *p* ≤ 0.05, ^**^p ≤ 0.01, ^***^p ≤ 0.001

The TC strategy group *Patient Communication and Care Management* emerged from factor and latent class analyses, in which Post-Discharge Care Consultation, Plain Language Communication at Home, Plain Language Communication at the Hospital, Symptom Management, and Transition Summary for Patients and Family Caregivers loaded onto the same factors or groups. Several of these strategies, or their close approximations, similarly bundled together in the retrospective study’s TC strategy groups (e.g., Symptom Management,^3^ Post-Discharge Care Coordination,^4^ and/or Transition Summary for Patients and Family Caregivers).

The TC strategy group *Hospital-Based Trust, Plain Language and Coordination* in part emerged from latent class analysis results, which showed that the TC strategy Identify High-Risk Patients and Intervene loaded with Medication Reconciliation and Transition Summary for Patients and Family Caregivers; the latter two TC strategies also grouped together in the retrospective study analysis. In addition, finite mixture modeling using hospital survey data supported the grouping of Post-Discharge Care Consultation, Medication Reconciliation, Plain Language Communication, and Transition Summary for Patients and Family Caregivers together. Based on factor and latent class results showing consistent affinity among Promote Trust at Hospital, Plain Language Communication at Hospital, and Post-Discharge Care Consultation, as well as the conceptual cohesion of these strategies, they were each included in the *Hospital-based trust, plain language and coordination* group.

The TC strategy group *Home-Based Trust, Plain Language and Coordination* emerged from latent class analysis (LCA) results and finite mixture model results in which Home visits, Referral to Community Services, Follow-up Appointment showed cohesion. In the LCA, Transition Team also loaded into the same class as these three TC strategies. LCA results also supported the combination of Plain Language Communication at Home and Promote Trust at Home, which conceptually, fit this TC strategy group’s emphasis on home-based post-discharge support activities. The retrospective study similarly identified a TC strategy group including Referral to Community Services and Transition Team.

The TC strategy group *Patient/Family Caregiver Assessment and Information Exchange Among Providers* emerged from results of both factor and latent class analyses in which the following TC strategies loaded together: Timely Exchange of Critical Patient Information Among Providers, Patient Goal/Preference Assessment, Identify High-Risk Patients and Intervene and Patient and Family Caregiver TC Needs Assessment. Two of these strategies (Timely Exchange of Critical Patient Information and Patient and Family Caregiver TC Needs Assessment) were similarly grouped in the retrospective study.

For the TC strategy group, *Assessment and Teach Back*, both factor and latent class analyses supported the grouping of Teach Back for Information and Skills, Language Assessment and Post-Discharge Care Consultation; evidence for grouping the latter two strategies also emerged from finite mixture model results. While none of these TC strategies were grouped in the retrospective study, analytically and conceptually these strategies showed cohesion pertaining to strategies employed by hospitals to increase adherence to patient care plans.

These series of analyses—complemented by expert review to ensure 1) consistency with prior ACHIEVE research (e.g., retrospective TC groups and patient and family caregiver focus group findings [25]), 2) consideration of service setting (e.g., hospital-based, bridging from hospital to home, or home-based), 3) practical relevance (e.g., sample size in each group must enable group comparison); and 3) minimal redundancy and overlap among groups— resulted in the groups as outlined in Table 6.

### Additional analytic confirmation

Random forest analyses were conducted as a confirmatory process to ascertain if machine learning methods would suggest additional important factors related to hospital readmissions by providing information about the relative “importance” of each variable in predicting 30-day readmissions (Table 10). Variables included in the random forest analysis were individual transitional care strategies, covariates (e.g., comorbidity, integrated health system affiliation), and the five newly defined groups of TC strategies. Results showed that, relative to other covariates and TC strategies, patient comorbidities and hospital cohort (e.g., medical, surgical, neurology) were most influential in predicting readmissions, accounting for a total of >200G^2^ and ~ 30% relative importance. Regarding the individual TC strategies and groups of TC strategies, those assessed through patient survey data (e.g., Helpful Healthcare Contact, Promote Trust, Plain Language Communication) were more important than the TC strategies assessed through the hospital survey data (e.g., Follow-up appointment, Teach Back for Information and Skills), with G^2^ ranging from 54 to 16, and relative importance from 7 to 2%. Importantly, random forest results did not suggest alternate groups nor an alternate approach to defining or classifying groups of TC strategies, with the key TC strategies from each group landing on the top of the importance tree. For example, Helpful Healthcare Contact has 7.2% relative importance, and Plain Language Communication at Home has 5.1% relative importance. As most information was used by splitting these two key TC strategies, the group including these two key TC strategies---*Patient Communication and Care Management* has only 2.1% relative importance. Notably, results were similar when we ran a boosted trees analysis, with patient comorbidity, Hierarchical Condition Category (HCC) cohort, and several patient-reported TC strategies among the most influential in the model for predicting 30-day hospital readmissions.

**Table 10:** Random Forest Analysis Results

Covariate	Number of Splits	G^**2**^	Relative Importance
Comorbidity	30,784	114.293	0.152
Hierarchical Condition Category (HCC) Cohort (e.g. Medical, Surgical, etc.)	29,459	106.811	0.142
Helpful Healthcare Contact	23,132	54.112	0.072
Promote Trust at Home	20,174	41.156	0.055
Symptom Management	21,791	38.865	0.052
Plain Language Communication at Home	19,470	38.479	0.051
Promote Trust at Hospital	17,908	36.913	0.049
Plain Language at Hospital	14,670	35.181	0.047
Integrated health system affiliation	6611	24.507	0.033
Referral to Community Services	11,620	19.863	0.026
Post Discharge Care Consultation	11,162	18.050	0.024
Patient and Family Caregiver TC Needs Assessment	10,775	17.161	0.023
Follow-up Appointment	6720	15.894	0.021
Home Visit	8808	15.822	0.021
TC Group: *Patient/Family Caregiver Assessment and Information Exchange*	9882	15.776	0.021
No TC Group	9707	15.496	0.021
TC Group: *Hospital-based Trust, Plain Language, and Coordination*	8540	15.482	0.021
TC Group: *Patient Communication and Care Management*	10,122	15.446	0.021
Teach Back for Information and Skills	9164	15.239	0.020
Timely Exchange of Critical Patient Information among Providers	7947	14.422	0.019
TC Group: *Home-based Trust, Plain Language and Coordination*	9593	14.227	0.019
Patient Goal/Preference Assessment	8617	13.815	0.018
Identify High-Risk Patients and Intervene	7320	12.338	0.016
Language Assessment	6329	11.422	0.015
Medication Reconciliation	5783	9.977	0.013
TC Group: *Assessment and Teach Back*	4770	8.429	0.011
Transition Team	4750	7.687	0.010
Transition Summary for Patients and Family Caregivers	3713	5.653	0.008

Notes:G^2^ = Likelihood Ratio Test Statistic, which is twice the [natural log] entropy, or twice the change in the entropy. Entropy is Σ -log(p) for each observation, where p is the probability attributed to the response that occurred. The G^2^ for a particular tree is the sum of the G^2^ values for each node that splits on that predictor. For a bootstrap forest model, the G^2^ values are added across the trees to give the G^2^ for that variable

HCC cohorts include: Medical, Surgical, Neurology, Cardiorespiratory, Cardiovascular

## Discussion

Project ACHIEVE aimed to provide practical, actionable guidance to hospitals searching for information about how to strategically invest in transitional care strategies in order to optimize patient outcomes. Prior systematic reviews found no individual strategy consistently associated with reduced readmissions, suggesting the need to evaluate TC strategies as implemented in groups or bundles [41]. Although numerous evidence-based, multifaceted transitional care models exist with evidence supporting their ability to reduce readmissions, [8, 9, 12, 32] adaptation of such models by implementation sites is common [23, 31, 42]. Little evidence exists regarding the question ‘Which transitional care strategies in which combination are most effective at improving patient outcomes in diverse populations and settings’? ACHIEVE capitalized on the natural experiment [26, 27] being conducted in the U.S. regarding the selective implementation of evidence-based transitional care models to answer this question. The present analysis describes the methodology for identifying the groups of TC strategies most commonly implemented by hospitals nationwide for subsequent evaluation of their comparative effectiveness. Our process resulted in five overlapping groups of TC strategies reflecting analytic and conceptual cohesion.

The five groups of TC strategies reported here that emerged from this process are being evaluated through the study’s prospective cohort analysis, the details of which will be reported elsewhere, to determine their detailed relationship with a broad spectrum of outcomes [43]. We believe the analyses that yielded these groups of TC strategies (See Table 5) provide methodological guidance for others seeking to conceptualize and evaluate complex interventions. We believe our findings provide a roadmap for the multistep, hybrid application of sophisticated analytic and conceptual techniques to categorize and define the ways in which TC strategies are naturally clustered so that these clusters (i.e., TC strategy groups) may then be evaluated.

Results of the sophisticated analyses conducted by the ACHIEVE research team have important practice implications. For example, one of the contextual analyses conducted, random forest analysis, demonstrated that patients’ underlying health conditions (e.g., comorbidities and hierarchical condition classification) were among the most important variables influencing readmissions, consistent with prior research [44–47]. This finding highlights the difficulties faced by hospitals and care providers attempting to improve transitional care. The next most important variables were implementation of TC strategies measured through survey of patients. This finding suggests that patient reporting of TC experience may be a more reliable indicator of TC implementation than hospital reporting for certain strategies—e.g., if caring and trust were fostered—given that implementation may vary by provider, care team, or circumstance. Patient survey data may more accurately reflect patients’ perception and experience more accurately reflecting impact of hospitals’ efforts. Another possible reason that patient-reported TC strategies exerted more influence over hospital readmissions may simply be that these strategies were meaningful components of discharge planning. Trust in one’s health care provider has emerged repeatedly in the literature as being associated with positive patient health outcomes [48], better care plan adherence [49], and patient satisfaction [50]. In fact, in focus groups and individual interviews conducted with 248 patients and family caregivers [25], participants voiced their strong desires to feel 1) cared for and about (Promote Trust), 2) prepared to implement the care plan (Plain Language Communication, Symptom Management), and 3) accountability on behalf of healthcare professionals regarding who to contact (Helpful Healthcare Contact).

Although the TC strategies in our model that were measured by patient survey were more important for predicting readmissions, strong rationale remains for measuring implementation of some TC strategies through hospital assessment. First, some TC strategies known to impact the quality of care do not have a patient-facing component, rendering it difficult for a patient to report on its appropriate application. For example, patients may not be aware if Medication Reconciliation was conducted by a designated pharmacist who contacted outpatient physicians or pharmacies, or whether their care team participates in Interdisciplinary Rounds. Second, some TC strategies are selectively applied based on risk stratification and are not universally appropriate for all patients. As most patients would screen negative for such interventions, their self-report of implementation may skew negative, even if the hospital appropriately implemented the strategy. Ultimately, we believe our hybrid approach of measuring implementation of certain TC strategies (e.g., Medication Reconciliation, Identify High Risk Patients and Intervene) from the hospital perspective, and measuring others (e.g., Plain Language Communication, Trust) from the patient perspective provides a more comprehensive assessment of implementation of TC strategies.

### Strengths and limitations

The hybrid approach outlined in Project ACHIEVE’s methodology—combining both analytic and conceptual methods— for defining groups of TC strategies for analysis represents a novel approach. The strengths of comprehensive, sophisticated analytic methods to identify patterns in how hospitals implement and patients experience transitional care efforts, which is important due to the wide variation in fidelity that hospitals report even when implementing evidence-based models for transitional care. The inclusion of expert interpretation and review to complement our analytic methods strengthens our approach, providing clinical experience and extensive research insight; an approach recommended when evidence informing clinical practice guidelines is inadequate [51]. By having clinical practitioners review the findings in context of their conceptual and practical relevance, we were able to ensure a degree of conceptual cohesion to enhance a TC strategy group’s relevance to real-world. Practice. By having expert researchers in transitional care also review the findings and inform groupings of TC strategies, we ensured there was sufficient distinction among the groups so that their comparative effectiveness could be evaluated.

While our approach of collecting data from both hospitals and patients is a strength, each source has its limitations. First, although most hospitals’ survey data^5^ were validated by a one to two-day site visit in which ACHIEVE researchers met with TC stakeholders across the hospital, the potential for self-report bias or incomplete implementation of certain strategies across all units or providers remains. Similarly, although our collection of some TC strategy data from the patient perspective is a strength, it has limitations. First, it is possible that patient outcomes following a care transition (e.g., readmission to hospital or emergency department) may have influenced patients’ perceptions of the transitional care strategies they received. The unfortunate CMS-imposed delay of patient survey administration to at least 51 days after hospital discharge may have exacerbated the potential for recall bias as well as selection bias. Sicker patients may have died or become less capable of participating. Further, patients with cognitive impairment were necessarily excluded due to their lack of consenting capacity, so our results may not apply as directly to that population. While our response rate of 57% for the patient survey compares favorably with similarly designed surveys—e.g., H-CAHPS surveys of patient experience typically average approximately 30% response rate [52]--the possibility remains that participants were systematically different than non-participants in ways that we were unable to measure. Because patients were initially recruited by hospitals, and not formally consented until contacted for the survey, we were unable to collect demographic information about those who did not consent. Therefore, we are unable to compare characteristics of participants with non-participants.

Finally, although our list of 22 transitional care strategies is extensive, and was rigorously developed through review of evidence-based TC models, review by an active and engaged Stakeholder Advisory Council and Scientific Advisory Council, and extensive structured discussions with the Project ACHIEVE research team [53], it is not exhaustive of all TC strategies used by hospitals nationwide, or even of the hospitals in our study. Some important TC practices used by hospitals may not have been included on our survey and therefore were likely omitted from study. Nevertheless, our findings provide practical guidance regarding a method of identifying patterns of patient exposure to bundles of TC strategies as a means of evaluating complex interventions.

## Conclusions

Our findings provide support that a data-driven approach using sophisticated statistical tools complemented by content experts can help identify underlying patterns of hospitals’ TC implementation efforts that correspond with better outcomes. Using such tools, this study identified five groups of TC strategies that have potential to improve patient outcomes.

## Supplementary Information

**Additional file 1.**

**Additional file 2.**

**Additional file 3.**

## Data Availability

The datasets used and/or analyzed during the current study are available from the corresponding author on reasonable request.

## Abbreviations

ACHIEVE: Achieving Patient-Centered Care and Optimized Health In Care Transitions by Evaluating the Value of Evidence
CMS: Centers for Medicare and Medicaid Services
ESRD: End Stage Renal Disease
FA: Factor analysis
FMM: Finite mixture model
HCAHPS: Hospital Consumer Assessment of Healthcare Providers and Systems
HCC: Hierarchical Condition Category
LCA: Latent class analysis
PCORI: Patient-Centered Outcomes Research Institute
Project BOOST: Better Outcomes by Optimizing Safe Transitions
Project RED: Re-Engineering Discharge
RF: Random Forest
SLMB: Specified Low-Income Medicare Beneficiary
TC: Transitional care
TCM: Transitional Care Model
U.S.: United States

## Footnotes

1 “Yes definitely” response was required for all TC strategies measured by the patient survey except for “Helpful Healthcare Contact”, in which the response categories were “Yes” versus “No”. See Table 3.

2 Hierarchical Condition Category (HCC) is a classification system designed to estimate healthcare costs for patients based on their risk level. HCC cohorts in our model include Medicine, Surgical, Cardiorespiratory, Cardiovascular, and Neurology.

3 Similar to Urgent Care Plan in the retrospective study

4 Similar to Care Coordination in the retrospective study

5 Site visits were conducted with 29 of the 42 participating hospitals. The 13 hospitals that were part of an integrated health care delivery system had previously participated in Phase 1 site visits and were thus, not repeated. Survey data for these hospitals were validated through follow-up phone calls with transitional care representative familiar with all sites and the system-level implementation of TC initiatives.
