# Supplementary material for: Improving evidence-based grouping of transitional care strategies in hospital implementation using statistical tools and expert review
Source: BMC Health Serv Res. 2021 Jan 7;21:35. doi: 10.1186/s12913-020-06020-9 (PMC7791839; doi:10.1186/s12913-020-06020-9)
Supplement: Supplementary file 1 — Additional file 1. [file 12913_2020_6020_MOESM1_ESM.docx]

Appendix 1. Hospital Survey


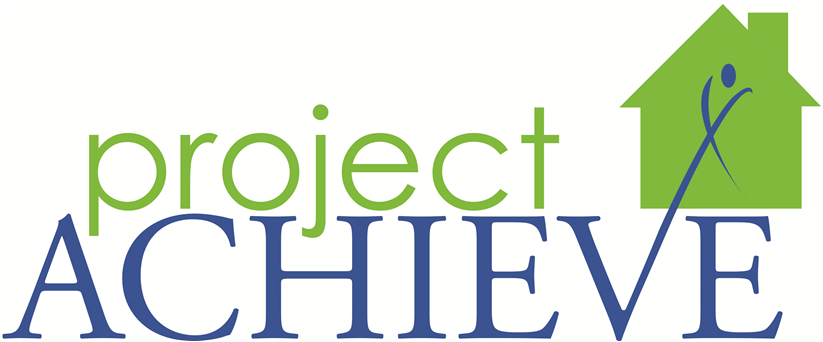


***A****chieving Patient-Centered****C****are and Optimized****H****ealth****I****n Care Transitions by* ***E****valuating the****V****alue of****E****vidence*

Organization Transitional Care Implementation Assessment Form
(OTCIA)

**Contents**

Part 1: Background………………………………………… ……………………1

Part 2: Transitional Care (TC) Efforts…………………………………….…..…..6

Part 3: Patient Assessment……………………………………………….......…..10

Part 4: Medication Reconciliation and Management…………………….….…..13

Part 5: Patient/Family Engagement and Education………………….…….....….15

Part 6: Transition Process…………………………………………….…....…….18

Part 7: Post-acute Care Linkages and Community Partnerships……………..….21

Part 8: Organizational Readiness for Implementing Change (ORIC)…. ……….24

This survey was developed as part of a research study funded through a Patient-Centered Outcomes Research Institute^®^ (PCORI^®^) Award (TC-1403-14049).

Organization Name: ____________________________________________________________

Your role in your organization:

1Quality Improvement, Quality Management, Performance Management

2Case Management/Care Coordination/Social Work/Discharge Planning

3Patient Safety Officer/Coordinator

4Other, Clinical Role, please specify: ______________________________________

5Other, Non-Clinical Role, please specify:__________________________________

6Other, please specify: __________________________________________________

**Part 1: Background**

1. Did your organization participate in or is your organization currently participating in (please check all that apply):

1CMS Community-based Care Transitions Program (CCTP)

2QIN-QIO (Quality Improvement Organization) community wide care transitions effort

3Hospital Engagement Network (HEN)

4Other regional and/or local care transition efforts

Please specify: __________________________________________________

5None

[If Q1=1 – 4, then 1a]

Q1a. If your organization is participating in one of the programs noted above, which organization serves as the primary lead of the community effort?

1 This hospital

2 Another hospital in this community

3 A post-acute provider (e.g., skilled nursing facility or home health agency)

4 A community-based organization or government agency (e.g., Health department, Area Agency on Aging, social services agency, faith-based organization, non-profit, or other community services provider)

5 Some other organization

6 Leadership is shared, or no leading agency identified

[If Q1=5, then 1b]

Q1b. If your organization does not participate in one of the above efforts, are you aware of any transitional care services offered by other organizations in your area?

1 No, I do not know of any TC services offered by other organizations in the

area

2 Services are offered by other organizations, but unsure of the nature of these

services

3 I know of services offered by other organizations, but these services are not

offered

in cooperation with services provided by this hospital

4This hospital works collaboratively with other organizations in the area to

coordinate TC services offered after discharge

1. Is your organization participating in any Medicare, Medicaid, and/or Commercial risk-based programs (please check all that apply):

1Accountable Care Organization (ACO)

2Bundled Payment on conditions that are included in CMS Hospital Readmission Reduction Program, including AMI, CHF, Pneumonia, COPD, elective total hip arthroplasty (THA) and total knee arthroplasty (TKA)

3Other risk-based contracts and/or innovation models, please specify: __________

4Other/Comments, please specify: _________________________

99Don’t know

1. Does your organization have a metrics tracking system/dashboard that includes readmissions and other relevant measures, e.g., emergency room visits post-hospital discharge, timeliness of discharge summary being completed, percent of patients discharged with a follow-up appointment?

1Yes

2No

99Don’t know

[If Q3=1, then 3a]

3a. With whom is this information shared? (please check all that apply)

1Internal staff

2Hospital Leadership

3Hospital Board of Directors

4Community Partners, such as SNF, home health agency, etc.

5Patient Family Advisory Council

6Public

7Other/Comments, please specify: ____________________________

1. Does your organization have some type of employee incentive program that includes performance on safety and quality outcomes or measures?

1Yes

2No

99Don’t know

[If Q4=1, then 4a]

4a. Who does this mainly effect at your organization?

1Leadership

2Most employees

3Other/Comments, please specify: _________________________

99Don’t know

1. Does your organization have a Patient Family Advisory Council (PFAC) or any type of hospital-wide patient/family advisory group?

1Yes

2No

99Don’t know

[If Q5=1, then 5a-5f]

5a. Does the PFAC or patient/family advisory group have a written mission?

1Yes

2No

99Don’t know

[If Q5a=1, then 5a.i.]

5a.i. Does the mission of the PFAC or patient/family advisory group include improving (please check all that apply):

1Patient safety

2Care transitions

3Patient experience

4Other/Comments, please specify:_________

99☐Don’t know

5b. To whom does the PFAC or patient/family advisory group report or communicate once they have recommendations or feedback to offer?

1Senior leadership at the hospital

2Staff liaison at hospital

3Hospital Board

4Other/Comments, please specify: ___________________________

99Don’t know

5c. Once the PFAC or patient/family advisory group makes their recommendations or provides feedback, does the PFAC or patient/family advisory group receive feedback on what the hospital did with that information? (please check all that apply)

*For example, “closing the loop” of communication by notifying the PFAC or patient/family advisory group of recommendations that resulted in a policy change, pilot program, or not being used at all and why.*

1Verbal feedback/reporting is provided

2Written feedback/reporting is provided

3Feedback/reporting is not provided

4Other types of feedback/Comments: __________________________

99Don’t know

5d. Is the PFAC or patient/family advisory group representative of the population your organization serves, taking into account diverse populations?

1Yes

2No

99Don’t know

5e. How is success of the PFAC or patient/family advisory group measured?

________________________________________________________________

5f. How does your hospital measure the effectiveness of the PFAC or patient/family advisory group?

1Meeting/Conference call attendance

2Existence or number of recommendations made

3Other/Comments, please specify: ______________________

99Don’t know

**Part 2: Transitional Care (TC) Efforts**

1. Which patient groups do you target in your organization’s TC efforts?
   (please check all that apply)

1☐All Patients

2☐Patients identified as high risk for readmission

3☐Patients with selected diagnoses (e.g., AMI, HF, COPD, etc.)

4☐Patients readmitted within the last 30 days

5☐Other/Comments, please specify: __________________________

99Don’t know

1. Are *most* of your organization’s transitional care efforts implemented in an integrated manner throughout your organization, e.g., a supervisory steering committee oversees, or active communication among the multiple teams/efforts to identify how each effort can complement the other?

1Yes

2No

99Don’t know

1. Are transitional care efforts implemented throughout your organization?

1☐All units of the organization implement transitional care efforts

2Most units implement transitional care efforts

3☐Only selected/disease-specific units implement transitional care efforts

4☐Other/Comments, please specify: ___________________________________

99Don’t know

[If Q8=3, then 8a]

8a. On which selected or disease/condition-specific units does your organization implement transitional care efforts? (please check all that apply)

| 1Cardiology | 4Respiratory Care |
| --- | --- |
| 2Oncology | 5Other, please specify:_____ |
| 3Medical/Surgical |  |

1. Are transitional care efforts implemented in collaboration with other organizations in your area?

1Yes

2No

99Don’t know

[If Q9=1, then 9a]

9a. Which of the following does your organization partner with to deliver transitional care services? (please check all that apply)

1Skilled nursing facilities

2Some health agencies

3Community-based organizations (eg. AAA, government service agencies,

non-profits, other community services)

4Other organizations (eg. Long term care providers, assisted living, hospice,

etc)

1. How long have transitional care improvement efforts been a part of your organization?

1☐>5 years

2☐4-5 years

3☐1-3 years

4☐Less than 1 year

99Don’t know

1. Please identify approximately when TC services *routinely* begin for patients discharged from your organization.

1At hospital admission

224-48 hours prior to discharge

3At discharge

4After discharge

5Other/Comments, please specify: _____________________________________

99Don’t know

1. Please identify for approximately how long patients *routinely* receive TC services from your organization.

1In hospital only

2One week following hospital discharge

3Less than one month but greater than one week following hospital discharge

4One month following hospital discharge

5No specific time frame; based on patient’s individual needs

6Other/Comments, please specify: __________________________

99Don’t know

1. Does your organization have a designated staff member who leads/manages the transitional care efforts at your organization?

1Yes

2No

99Don’t know

1. Does your organization have a designated team that facilitates the implementation of transitional care efforts at your organization?

1☐Yes

2☐No

99Don’t know

[If Q14=1, then 14a]

14a. The designated team includes: (please check all that apply)

| Senior management of the hospital | Social Workers |
| --- | --- |
| Physicians | Navigators (can be lay navigators, in addition to nurses or social workers) |
| Advanced practice nurses | Quality improvement staff |
| Physician Assistants | Pharmacists |
| Nurses | Therapists/Counselors (e.g., psychologist or psychiatrist) |
| Case managers | PT/OT/ST |
| Other, please specify: |  |

1. What types of communication methods are used among members in your organization to communicate about patients’ discharges or transitional care needs?

1☐Phone call or pager

2☐Electronic Health Record (EHR)

3Secure email

4☐Designated Interdisciplinary Rounds/Huddles/Meetings

5Videophone, Skype or FaceTime

6☐Other/Comments, please specify: ____________________________________

99Don’t know

[If Q15=4, then 15a]

15a. Are patients and/or family members present at the Interdisciplinary

Rounds/Huddles/Meetings?

1Yes

2No

99Don’t know

1. What is the structured process your members use in communicating? (please check all that apply)

1Checklist(s)

2SBAR (Situation, Background, Assessment, Recommendation)

3Other/Comments, please specify:_____________________

99Don’t know

**Part 3: Patient Assessment**

1. Does your organization use a protocol/risk assessment tool to identify patients who are at high risk of readmission or have high-risk scenarios that could potentially result in poor outcomes?

1  Yes 2  No 99  Don’t know

[If 17=1, then 17a-17b]

17a. Which protocol/risk assessment tool does your organization *routinely* use? (please check all that apply)

1☐LACE

2☐BOOST 8Ps

3☐Home-grown analytic tool (a tool that your own organization created)

4Specific surveys

5☐Other/Comments, please specify: __________________________

99Don’t know

17b. To what extent does your organization *consistently* use a protocol/risk assessment tool to identify patients in need of transitional care services?

| To a great extent | Somewhat | Very little | Not at all | Don’t know |
| --- | --- | --- | --- | --- |
| 1 | 2 | 3 | 4 | 99 |

1. When does the risk assessment begin?

1☐At admission to the organization

2☐Sometime during the hospitalization/Prior to discharge date

3☐Day of discharge

4☐Other/Comments, please specify: __________________________

99Don’t know

1. Which of the following criteria are used by your organization to identify patients in need of transitional care services?

| Certain Diagnoses or Comorbidities | Lack of social support (consistent caregiver, transportation, etc.) |
| --- | --- |
| Problems with medications (Polypharmacy and/or high-risk medication such as anticoagulants) | Emotional/Psychological status (Depression, Anxiety, etc.) |
| Use of hospital/emergency department within last 30 days | History of Mental Health/Behavioral Health issues |
| Use of hospital/emergency department within last 90 days/3 months | Substance Use (History, current use or inappropriate use of alcohol, prescription medications, or illicit drugs) |
| Socioeconomic status (e.g., financial issues, homelessness, etc.) | Cognitive impairment |
| Limited/Poor health literacy | Limitations with physical functioning (e.g., frailty, deconditioning, unable to perform own ADLs) |
| Language barriers | Other/Comments, please specify: _________________ |

1. Who *typically* identifies patients in need of transitional care services? (please check the primary staff member- only one answer)

1Admitting nurse

2Admitting doctor

3Nurse at hospital

4Nurse from outside an organization

5Attending doctor

6Social worker at hospital

7Social worker from an outside organization

8Hospital administration

9Case manager at hospital

10Case manager from an outside organization

11Designated staff member responsible for transitional care/Transitional Care Coordinator/Discharge planner at hospital

12Designated staff member responsible for transitional care/Transitional Care Coordinator/Discharge planner from an outside organization

13Other/Comments, please specify:_______________________________________

99Don’t know

1. To what extent does your organization implement risk-specific interventions tailored to a patient's individual risk of poor outcomes or other post-discharge adverse event (e.g., referral to community services or outpatient case managers for patients with psychosocial issues)?

| To a great extent | Somewhat | Very little | Not at all | Don’t know |
| --- | --- | --- | --- | --- |
| 1 ☐ | 2 ☐ | 3 ☐ | 4 ☐ | 99 |

**Part 4: Medication Reconciliation and Management**

1. Medication history is *initially* obtained by: (please check all that apply)

1☐Emergency department staff

2☐Admitting medical team

3☐Pharmacist or pharmacy technician

4☐Nurses

5No designated person

6☐Other/Comments, please specify:__________________________________

99Don’t know

1. How often are contacts made with outside pharmacies and/or primary care providers for clarifying a patient’s current medication list when needed (i.e., medication reconciliation)?

1☐Always

2☐Usually

3☐Sometimes

4☐Never

99Don’t know

1. Is there a designated person responsible for conducting medication reconciliation at discharge?

1Yes

2No

99Don’t know

[If Q24=1, then 24a]

24a. Who is the designated person *most often* responsible for conducting medication reconciliation at discharge?

1☐Discharging physician, physician assistant or nurse practitioner

2☐Nurse

3☐Pharmacist

4☐Responsibility is not formally assigned

5Other/Comments, please specify:__________________________________

99Don’t know

1. What tools are in place to facilitate medication reconciliation at your organization?
   (please check all that apply)

1☐Paper-based standardized form

2☐Web-based tool

3☐Form/tool built into Electronic Medical Record (EMR)

4☐No standardized form or tool is used for medication reconciliation

5Other/Comments, please specify: ____________________________________

99Don’t know

1. As part of the discharge process, does staff or a designated staff person *routinely* ask patients whether they can afford their medications?

1☐Yes, for all patients

2☐Yes, for some patients depending on the medication(s) or their circumstances

3☐No, not routine

4Other/Comments, please specify: ______________________________________

99Don’t know

1. How often are your patients discharged from the hospital with any new medications in hand?

1☐Always

2☐Usually

3☐Sometimes

4☐Never

99Don’t know

**Part 5: Patient/Family Engagement and Education**

1. Please identify which assessment/planning services are currently utilized at your organization:

| **Assessment/Planning Services** | Yes  (1) | No  (2) |
| --- | --- | --- |
| Assessment of patient's transitional care needs using explicit criteria |  |  |
| Identification of patient's health goals and preferences |  |  |
| Assessment of patient's learning capability and style |  |  |
| Identification of family caregiver |  |  |
| Assessment of family caregiver's transitional care needs (using explicit criteria) |  |  |
| Engagement of patient and family caregivers in decision making (i.e., consider patient and caregiver input while making decisions) |  |  |

Teach Back is one method that health care providers use to ensure information is explained clearly to patients and their caregivers. It includes asking a patient or caregiver, in their own words, what they need to know, explaining information clearly or demonstrating a skill, and checking to make sure they understand or can demonstrate the skill themselves.

1. Is the Teach Back Method used formally in your organization?

1Yes

2No

99Don’t know

[If Q29=1, then 29a-29b]

29a. Which patients and their families/caregivers *regularly* receive the Teach Back Method at your organization?

1☐All patients/families

2☐Some patients and their families depending on their diagnosis and/or circumstances (e.g., elderly patients or non-English speaking patients to ensure comprehension)

3☐Patients on certain floors/units

4Other/Comments, please specify:_____________________________

99☐Don’t know

29b. Is Teach Back an expected competency for nurses at your hospital?

1Yes

2No

99Don’t know

1. Does your organization provide opportunities that allow patients and families/caregivers to learn new information or skills needed for self-care at home:

1Yes

2No

99Don’t know

[If Q30=1, then 30a-30c]

30a. How does your organization provide opportunities that allow patients and family/caregivers to learn new information or skills needed for self- care at home: (please check all that apply)

1Teach Back Method

2Group classes

3One-on-one coaching

4A structured educational program

5Other/Comments, please specify:________________________

99Don’t know

30b. How does your organization assess skills competency of the patient and/or family/caregiver? (please check all that apply)

1Patient and/or family/caregiver can verbalize new skills/information

2Patient and/or family/caregiver demonstrates the new skill

3Other/Comments, please specify:______________________

99Don’t know

30c. To what extent does your organization provide opportunities that allow patients and family/caregivers to practice new skills needed for self-care:

| To a great extent | Somewhat | Very little | Not at all | Don’t know |
| --- | --- | --- | --- | --- |
| 1 | 2 ☐ | 3 ☐ | 4 ☐ | 99 |

31. To what extent does your organization *consistently* use “plain language” for patient and family/caregiver communication:

| To a great extent | Somewhat | Very little | Not at all | Don’t know |
| --- | --- | --- | --- | --- |
| 1☐ | 2☐ | 3☐ | 4☐ | 99 |

32. To what extent does your organization *consistently* identify, communicate and offer interpreter service to patients who need it:

| To a great extent | Somewhat | Very little | Not at all | Don’t know |
| --- | --- | --- | --- | --- |
| 1☐ | 2☐ | 3☐ | 4☐ | 99 |

33. To what extent does your organization *consistently* provide educational materials in the language that patients prefer, if patients are non-English speaking:

| To a great extent | Somewhat | Very little | Not at all | Don’t know |
| --- | --- | --- | --- | --- |
| 1☐ | 2☐ | 3☐ | 4☐ | 99 |

34. To what extent does your organization provide a patient-centered transition record or personal health record (e.g., list of diagnoses, allergies, medications, physicians, contact information) to patients/caregivers:

| To a great extent | Somewhat | Very little | Not at all | Don’t know |
| --- | --- | --- | --- | --- |
| 1☐ | 2☐ | 3☐ | 4☐ | 99 |

35. Does your organization use non-clinical laypersons or volunteers -- such as community health workers, non-health professionals, peer educators, or lay health advisors/navigators -- to follow up with patients after discharge?

1☐Yes, hospital employs or contracts with these workers

2☐Yes, hospital uses volunteer workers

3☐Yes, hospital refers patients to workers employed and/or managed by an outside organization

4☐No, our organization does not use non-clinical laypersons or volunteers

5Other/Comments, please specify:____________________________

99Don’t know

**Part 6: Transition Process**

36. For patients receiving transitional care services, on the day of discharge, do patients leave the hospital with an outpatient follow-up appointment already arranged?

1☐Always

2☐Usually

3☐Sometimes

4☐Never

99Don’t Know

37. To what extent does your organization *routinely* make referrals and/or arrangements for community-based services? (e.g., transportation assistance, Meals on Wheels, etc.)

| To a great extent | Somewhat | Very little | Not at all | Don’t know |
| --- | --- | --- | --- | --- |
| 1☐ | 2☐ | 3☐ | 4☐ | 99 |

38. To what extent does your organization *routinely* use a specific transition team (i.e., care coordination) to coordinate transitional care plans across hospital and post-home sites of care?

| To a great extent | Somewhat | Very little | Not at all | Don’t know |
| --- | --- | --- | --- | --- |
| 1☐ | 2☐ | 3☐ | 4☐ | 99 |

39. Is there a reliable process in place to ensure outpatient care providers (i.e., primary care physicians) are alerted to the patient’s hospital *admission* within 24 hours of admission?

1. ☐ Yes 2 ☐ No 99 Don’t know

40. Does your organization use a standardized template for discharge summaries?

1☐Yes

2☐No

99Don’t Know

41. How quickly is a patient’s discharge summary typically completed and available for viewing in the EMR or printed on paper?

1☐At Discharge

2☐Within 48 hours

3☐Within 72 hours

4☐Within 7 days (1 week)

5Within 14 days (2 weeks)

6☐Within 30 days (1 month)

7No explicit goals/policies defining a time-frame for completing the discharge summary

8Other/Comments, please specify:____________________________

99Don’t know

42. For what proportion of patients is a paper or electronic discharge summary sent directly to the patient’s primary care providers or post-acute providers such as nursing homes/SNFs, home health agencies, etc.?

1☐All

2☐Most

3☐Some

4☐None

99Don’t know

43. For what proportion of patients do outpatient care and community service providers have access to inpatient electronic records?

1☐All

2☐Most

3☐Some

4☐None

99Don’t know

44. At the time of hospital discharge, for what proportion of patients are their goals and preferences (e.g., Goals of Care or DNR status) communicated to primary care providers or post-acute providers such as nursing homes/SNFs, home health agencies, etc.?

1☐All

2☐Most

3☐Some

4☐None

99Don’t know

45. Is there someone within your organization assigned to follow up on test results that return after the patient is discharged?

1 ☐ Yes 2 ☐ No 99 Don’t know

**Part 7: Post-acute Care Linkages and Community Partnerships**

46. For what proportion of patients receiving transitional care services does your organization regularly call after discharge to either follow up on post-discharge needs or to provide additional education?

1☐All

2☐Most

3☐Some

4None

99Don’t know

[If Q46=1, 2, or 3; then 46a]

46a. When does your organization regularly call patients after discharge to either follow up on post-discharge needs or to provide additional education?

1☐Within 48 hours of discharge

2Within 72 hours of discharge

3☐Within 1 week of discharge

4☐Within 2 weeks of discharge

5☐We do not call patients regularly after discharge

6Other/Comments, please specify: ______________________

99Don’t know

47. For what proportion of patients receiving transitional care services does your hospital or a community based organization (e.g., Area Agency on Aging) conduct home visits after discharge, either by a care coordinator or equivalent?

1☐All

2☐Most

3☐Some

4None

99Don’t know

48. For what proportion of patients does your hospital arrange telemonitoring after discharge?

1☐All

2☐Most

3☐Some

4☐None

99Don’t know

49. Does your hospital use telemedicine/telehealth technology to care for patients’ post-hospital discharge?

1 ☐ Yes 2 ☐ No 99  Don’t know

[If Q49=1, then 49a]

49a. In which setting do you use this to deliver care post-acute? (please check all that apply)

1☐Home

2Home Health

3☐Skilled Nursing Facility

4☐Other/Comments, please specify:_________________________________

99Don’t know

50. With which of the following post-acute care sites and/or community based organizations has your organization partnered with to improve care transitions and prevent poor outcomes? (please check all that apply)

1Community home health care agencies

2Skilled nursing facilities

3Long Term Acute Care (LTAC) Hospitals

4Independent Rehabilitation Facilities

5Palliative care and hospice providers

6Community physicians or physician groups

7Community based organization (Area Agency on Aging, faith-based organization, etc.)

8Other local hospitals

9Local or state health department

10Fire department/EMS

11Other state or regional agencies
12Other/Comments, please specify: ____________________

51. For patients discharged to skilled nursing facilities or with home health services, does your organization provide direct contact information for a specific inpatient physician to contact in case of questions?

1☐Always

2☐Usually

3☐Sometimes

4☐Never

99Don’t know

**Part 8: Organizational Readiness for Implementing Change (ORIC)**

For the following statements, “this change” refers to “transitional care processes or strategies.” Please indicate your level of agreement with the following statements.

52. People who work here feel confident that the organization can get people invested in implementing this change.

1Disagree

2Somewhat Disagree

3Neither Agree nor Disagree

4Somewhat Agree

5Agree

53. People who work here are committed to implementing this change.

1Disagree

2Somewhat Disagree

3Neither Agree nor Disagree

4Somewhat Agree

5Agree

54. People who work here feel confident that they can keep track of progress in implementing this change.

1Disagree

2Somewhat Disagree

3Neither Agree nor Disagree

4Somewhat Agree

5Agree

55. People who work here will do whatever it takes to implement this change.

1Disagree

2Somewhat Disagree

3Neither Agree nor Disagree

4Somewhat Agree

5Agree

56. People who work here feel confident that the organization can support people as they adjust to this change.

1Disagree

2Somewhat Disagree

3Neither Agree nor Disagree

4Somewhat Agree

5Agree

57. People who work here want to implement this change.

1Disagree

2Somewhat Disagree

3Neither Agree nor Disagree

4Somewhat Agree

5Agree

58. People who work here feel confident that they can keep the momentum going in implementing this change.

1Disagree

2Somewhat Disagree

3Neither Agree nor Disagree

4Somewhat Agree

5Agree

59. People who work here feel confident that they can handle the challenges that might arise in implementing this change.

1Disagree

2Somewhat Disagree

3Neither Agree nor Disagree

4Somewhat Agree

5Agree

60. People who work here are determined to implement this change.

1Disagree

2Somewhat Disagree

3Neither Agree nor Disagree

4Somewhat Agree

5Agree

61. People who work here feel confident that they can coordinate tasks so that implementation goes smoothly.

1Disagree

2Somewhat Disagree

3Neither Agree nor Disagree

4Somewhat Agree

5Agree

62. People who work here are motivated to implement this change.

1Disagree

2Somewhat Disagree

3Neither Agree nor Disagree

4Somewhat Agree

5Agree

63. People who work here feel confident that they can manage the politics of implementing this change.

1Disagree

2Somewhat Disagree

3Neither Agree nor Disagree

4Somewhat Agree

5Agree
