# Supplementary material for: Improving evidence-based grouping of transitional care strategies in hospital implementation using statistical tools and expert review
Source: BMC Health Serv Res. 2021 Jan 7;21:35. doi: 10.1186/s12913-020-06020-9 (PMC7791839; doi:10.1186/s12913-020-06020-9)
Supplement: Supplementary file 2 — Additional file 2. [file 12913_2020_6020_MOESM2_ESM.docx]

Appendix 2:

English Patient Survey, Mail Version

This survey was developed as part of a research study funded through a Patient-Centered Outcomes Research Institute^®^ (PCORI^®^) Award (TC-1403-14049).

INTRODUCTION

1. Our records show that you had the following hospital stay at:

[HOSPITAL NAME]

Date you left the hospital:

[DISCHARGE DATE]

Is that correct?

Yes

No 🡪 Go to #49

This survey asks about that hospital stay and your experiences since you’ve been home.

IN THE HOSPITAL

When answering these questions, please only think about your hospital stay named in Question 1.

1. In this survey, a healthcare professional means a doctor, nurse, pharmacist, physical or occupational therapist, social worker, or case manager.

In the hospital, did any healthcare professionals tell you or show you what you needed to do to take care of yourself at home?

Yes, definitely

Yes, somewhat

No

You already knew what to do

1. In the hospital, did any healthcare professionals make sure you understood what you needed to do to take care of yourself at home?

Yes, definitely

Yes, somewhat

No

1. In the hospital, did you practice the things you would need to do at home?

Yes, definitely

Yes, somewhat

No

You did not need to practice

anything

1. In the hospital, did healthcare professionals explain things in a way you could understand?

Yes, definitely

Yes, somewhat

No

1. In the hospital, did healthcare professionals seem to care about you as a person?

Yes, definitely

Yes, somewhat

No

1. In the hospital, did you trust healthcare professionals’ judgments about your medical care?

Yes, definitely

Yes, somewhat

No

1. Before you left the hospital, did you get information about what symptoms to look out for at home?

Yes, definitely

Yes, somewhat

No

1. Before you left the hospital, did any healthcare professionals talk with you about prescription or over-the-counter medicine you should take at home?

Yes, definitely

Yes, somewhat

No 🡪 Go to #11

You did not have to take any

medicine at home 🡪 Go to #11

1. Before you left the hospital, were the possible side effects of each medicine clear to you?

Yes, definitely

Yes, somewhat

No

1. Before you left the hospital, you should have received written information about how to take care of yourself at home. How helpful was the information?

Very helpful

Moderately helpful

A little helpful

Not at all helpful

You did not receive or did not

read the information

11a. [SPANISH VERSION ONLY] Was the written information in Spanish?

Yes

No

1. Before you left the hospital, did you have a doctor’s appointment scheduled?

Yes

No

1. On the day you left the hospital, did you think it was too soon for you to leave?

Yes, definitely

Yes, somewhat

No 🡪 Go to #15

1. Was one of the reasons it was too soon because you needed more care than could be provided at home?

Yes

No

SINCE YOU’VE BEEN HOME

The next questions ask about the care you’ve received since you’ve been home from the hospital stay named in Question 1.

1. Since you’ve been home, have you had contact information for a healthcare professional you could reach out to if you had any problems or questions?

Yes

No 🡪 Go to #17

1. If you tried to contact them, did you get help with your problems or questions?

Yes, definitely

Yes, somewhat

No

You did not try to contact them

1. Since you’ve been home, have you had to take any prescription or over-the-counter medicines?

Yes

No 🡪 Go to #19

1. Since you’ve been home, has there been a time when you did not take your medicine as directed?

Yes

No

1. Since you’ve been home, have you needed to use any medical supplies or equipment—like bandages, a blood pressure monitor, oxygen, or a walker?

Yes

No 🡪 Go to #21

1. Since you’ve been home, how well have you been able to use your medical supplies or equipment?

Very well

Moderately well

Slightly well

Not at all well

You have not used your medical

supplies or equipment

1. Since you’ve been home, have you had to take care of a wound or surgical site, including a catheter or ostomy?

Yes

No 🡪 Go to #23

1. Since you’ve been home, how well have you been able to take care of your wound or surgical site?

Very well

Moderately well

Slightly well

Not at all well

1. Since you’ve been home, have you received transportation assistance from a service for older adults or people with disabilities?

Yes 🡪 Go to #25

No

1. Did you want transportation assistance from a service for older adults or people with disabilities?

Yes

No

1. Since you’ve been home, have you received meals delivered to your home from a service for older adults or people with disabilities?

Yes 🡪 Go to #27

No

1. Did you want meals delivered to your home from a service for older adults or people with disabilities?

Yes

No

1. Since you’ve been home, have you received physical or occupational therapy services?

Yes 🡪 Go to #29

No

1. Did you need physical or occupational therapy services?

Yes

No

1. A healthcare professional means a doctor, nurse, pharmacist, physical or occupational therapist, social worker, or case manager.

Since you’ve been home, have any healthcare professionals come to your home?

Yes 🡪 Go to #31

No

1. Did you want home visits from a healthcare professional?

Yes

No

1. Since you’ve been home, have you talked with or communicated with any healthcare professionals?

Yes

No 🡪 Go to #37

1. Since you’ve been home, have healthcare professionals helped you manage any changes or unexpected problems with your care?

Yes, definitely

Yes, somewhat

No

You’ve had no changes or

unexpected problems with your care

1. Since you’ve been home, have healthcare professionals explained things in a way you could understand?

Yes, definitely

Yes, somewhat

No

1. Since you’ve been home, have healthcare professionals seemed to care about you as a person?

Yes, definitely

Yes, somewhat

No

1. Since you’ve been home, have you trusted healthcare professionals’ judgments about your medical care?

Yes, definitely

Yes, somewhat

No

1. Since you’ve been home, has a healthcare professional told you something that went against what another healthcare professional told you?

Yes, definitely

Yes, somewhat

No

OVERALL QUALITY OF YOUR CARE

1. Overall, how would you rate the hospital in preparing you to take care of yourself at home?

Excellent

Very good

Good

Fair

Poor

1. Overall, how would you rate your ability to take care of yourself since you’ve been home?

Excellent

Very good

Good

Fair

Poor

1. Overall, how would you rate the care you have received from healthcare professionals since you’ve been home?

Excellent

Very good

Good

Fair

Poor

You have not received care from

any healthcare professionals since you’ve been home

1. Overall, have healthcare professionals been there for you as much as you needed?

Yes, definitely

Yes, somewhat

No

**YOUR OVERALL HEALTH**

1. In the past week, how would you rate your physical health?

Excellent

Very good

Good

Fair

Poor

1. In the past week, how would you rate your mental or emotional health, including your mood and your ability to think?

Excellent

Very good

Good

Fair

Poor

1. In the past week, how would you rate your sleep?

Excellent

Very good

Good

Fair

Poor

1. In the past week, how often have you had bodily pain?

Not at all

Once in the past week

Several times in the past week

Daily

All the time

1. In the past week, to what extent were you able to carry out your everyday physical activities such as walking, climbing stairs, carrying groceries, or moving a chair?

Completely

Mostly

Moderately

A little

Not at all

**A FAMILY MEMBER OR FRIEND WHO HELPED YOU AT HOME**

1. Since you’ve been home, has a family member or friend, who is 18 or older, helped you take care of yourself?

Yes

No 🡪 Go to #49

1. Who provided the most help? Mark one.

Your husband/wife

Your boyfriend/girlfriend

Your partner/significant other

Your son/daughter (include in-laws)

Your brother/sister (include in-laws)

Your father/mother (include in-laws)

Your grandson/granddaughter

Other relative

A friend

Someone else

1. Please provide contact information for the family member/friend from Question 47 so we can ask them about the care you’ve received. This information will be kept confidential.

Family member or friend’s first name: __________________

Family member or friend’s last name: __________________

Their phone #: ____________________

ABOUT YOU

1. How confident are you in filling out medical forms by yourself?

Extremely confident

Quite a bit confident

Somewhat confident

A little confident

Not at all confident

1. Do you usually ask someone to help you read materials you receive from the hospital?

Yes

No

1. Are you male or female?

Male

Female

1. What is the highest grade or level of school you have completed?

8^th^ grade or less

Some high school, but did not

graduate

High school graduate or GED

Some college or 2-year degree

4-year college graduate

More than a 4-year college

degree

1. Are you of Hispanic, Latino, or Spanish origin?

Yes, Hispanic, Latino, or Spanish

No, not Hispanic, Latino, or

Spanish

1. What is your race?

Mark all that apply.

White

Black or African American

Asian

Native Hawaiian or other Pacific

Islander

American Indian or Alaska Native

Other

1. Did someone help you complete this survey?

Yes

No 🡪 Thank you. Please return

your completed survey.

1. How did that person help you? Mark all that apply.

Read the questions to me

Wrote down the answers I gave

Answered the questions for me

Translated the questions into my

language

Helped in some other way

Thank you.
Please return your completed survey.
