# Supplementary material for: Improving evidence-based grouping of transitional care strategies in hospital implementation using statistical tools and expert review
Source: BMC Health Serv Res. 2021 Jan 7;21:35. doi: 10.1186/s12913-020-06020-9 (PMC7791839; doi:10.1186/s12913-020-06020-9)
Supplement: Supplementary file 3 — Additional file 3. [file 12913_2020_6020_MOESM3_ESM.docx]

| **Table 1. TC Strategy Groups Emerging from Project ACHIEVE Retrospective Analysis** | |
| --- | --- |
| **TC Group** | **Required TC Strategies** |
| **Care Plan** | - Urgent Care Plan ^b^   **AND**   - Care Coordination^c^  **OR** Transition Summary for Patients and Family Caregivers |
| **Shared Decision** | - Shared Decisions   **AND**   - Patient and Family Caregiver TC Needs Assessment **OR** Teach Back |
| **Identify High-Risk** | - Identify High-Risk Patients and Intervene **OR** Referral to Community Services   **AND**   - Transition Team **OR** Interdisciplinary Approach |
| **Medication Reconciliation** | - Medication Reconciliation **OR** Follow-up Appointment   **AND**   - Transition Team **OR** Transition Summary for Patients and Family Caregivers |
| **Cross-Setting Information Exchange** | - Timely Exchange of Critical Patient Information Among Providers   **AND**   - Patient and Family Caregiver TC Needs Assessment **OR** Teach Back |
| ^a^ Hospitals could be in more than one TC group.  ^b^ “Urgent Care Plan” TC strategy in retrospective study is conceptually similar to “Helpful Healthcare Contact” and “Symptom Management” in prospective study  ^c^ “Care Coordination” TC strategy in retrospective study is conceptually similar to “Post Discharge Care Consultation in prospective study. | |
